# Ribosome deficiency induces *Salmonella* filamentation within host cells

**DOI:** 10.1128/mbio.01417-25

**Published:** 2025-06-30

**Authors:** Zhihui Lyu, Cierra Wilson, Kalyn Weiss, Spencer Lewis, Kurt Fredrick, William Margolin, Jiqiang Ling

**Affiliations:** 1 Department of Cell Biology and Molecular Genetics, The University of Maryland, College Park, Maryland, USA; 2 Department of Microbiology and Molecular Genetics, McGovern Medical School, The University of Texas Health Science Center 12340, Houston, Texas, USA; 3 Department of Microbiology, The Ohio State University 215854 , Columbus, Ohio, USA; University of Georgia Center for Food Safety, Griffin, Georgia, USA

**Keywords:** translational defect, ribotoxic stress, bacteria-host interactions, stress adaptation, cell division

## Abstract

The ribosome is the central hub for protein synthesis and is heavily targeted by antibiotics. Ribosomal mutations, antibiotic treatment, and nutrient starvation can alter translational efficiency and lead to stressed cells. Ribosome deficiency plays a critical role in stress responses and disease progression; yet, how it affects bacteria-host interactions remains poorly understood. In this study, we show that a ribosome-deficient strain exhibits a surprising morphological change from rod shape to filamentous in *Salmonella* cells growing inside host macrophages. Such filamentation depends on an acidic condition within macrophages and in a defined medium mimicking macrophage conditions. Further genetic analyses revealed that filamentation of the ribosome-deficient strain depends on overexpression of *hisH*, a gene involved in histidine biosynthesis. Transcription of the histidine biosynthesis operon is regulated by a small leader peptide HisL. Slow translation of HisL in the mutant strain activates transcription of the histidine operon and induces filamentation. In support of this model, we show that ribosome inhibitors also increase the expression of the histidine operon and cause filamentation in wild-type *Salmonella*. Bacterial filamentation has been implicated as an adaptive strategy. We show that filamentation improves the survival of *Salmonella* cells under acid stress, and filamentous cells resume normal division after the acid stress is removed. Our work thus demonstrates that ribosome deficiency caused by mutations and antibiotics induces *Salmonella* filamentation in host cells as a potential survival strategy.

**IMPORTANCE:**

Bacteria growing inside host cells encounter various stresses and have evolved multiple adaptive mechanisms. One such mechanism is morphological changes, such as from rod-shaped cells to filaments. *Salmonella* is a rod-shaped pathogen that infects over 100 million people each year as well as numerous farmed animals. In this work, we present new findings that slowing down protein synthesis causes *Salmonella* to filament inside mammalian host cells. Combining genetic, molecular, and cell biology approaches, we demonstrate that filamentation of Salmonella cells is caused by translational and transcriptional regulation of the histidine operon. Filamentous cells appear to tolerate acid stress better and resume cell division after the stress is removed. This work highlights intriguing translational control of bacterial cell division and morphology, which may facilitate *Salmonella* cells to adapt to the host environment.

## INTRODUCTION

Protein synthesis is a central demand for all cells and consumes over 50% of cellular energy (1–3). During translation, aminoacyl-tRNA synthetases (aaRSs) selectively attach each amino acid to the corresponding tRNA, and the resulting aminoacyl-tRNAs (aa-tRNAs) are delivered to the ribosome by elongation factors (EF-Tu in bacteria and EF1A in eukaryotes) (4–8). Translation is highly regulated and interconnects with other cellular processes. In eukaryotes, ribosome stalling due to defective translation activates the ribotoxic and integrated stress responses, which play important roles in human diseases such as cancer and neurological disorders (9–11). In bacteria, amino acid starvation leads to ribosome pausing and production of the alarmone (p)ppGpp, which activates the stringent response (12, 13). In addition to nutrient starvation, ribosome pausing can also result from antibiotics and mutations in the translation machinery, leading to attenuation of global translation and cellular stress (14–16). How such ribosome deficiency affects the survival and adaptation of bacteria in the host remains poorly understood.

Filamentation is a specific consequence of a block to cell division of rod-shaped bacteria (17–19). Cell division mediated by the divisome is crucial for bacterial proliferation (17). Bacterial filamentation is induced by host-cell environments, mutations, and stress conditions and is considered a survival strategy under host and antimicrobial stress conditions (19–23). *Escherichia* and *Salmonella* are rod-shaped Gram-negative Enterobacteriaceae that include multiple species of important pathogens, such as *S. enterica* and *E. coli*. Filamentation of uropathogenic *E. coli* (UPEC) occurs during infections and facilitates its attachment to host cells (21, 24). Filamentation in *S. enterica* is also known to be induced by the host environment (25–28), fatty acids (29), low temperature (30), and osmotic stress (31). Earlier studies have shown that the *S. enterica* Typhimurium strain SL1344 is filamentous within macrophages and non-phagocytic eukaryotic cells (25, 27, 28). Filamentation of SL1344 depends on a mutation in the *hisG* gene, which is required for the first step of histidine synthesis, and other unknown factors in the cell (26).

In this work, we demonstrate that translation stress resulting from ribosomal mutations or ribosome inhibitors causes a filamentation phenotype in *Salmonella* during growth inside macrophage cells. The wild-type (WT) *S. enterica* Typhimurium strain ATCC 14,028 s exhibits normal cell division within macrophages, but a K42N mutation in the ribosomal protein uS12 (encoded by *rpsL*) induces *Salmonella* filamentation in host cells. Mutations in the *rpsL* gene are frequently identified in streptomycin (Str)-resistant bacterial strains and often lead to slower ribosomes (32–37). We show that filamentation of *rpsL* K42N (rpsL*) *Salmonella* depends on overexpression of the histidine operon resulting from a translational defect of the small leader peptide HisL. Deleting *hisH* within the histidine operon abolished filamentation of rpsL* cells, and overexpressing *hisH* is sufficient to induce filamentation in WT *Salmonella. In* addition to *Salmonella*, E. coli rpsL* cells also filament under acidic conditions. We further show that adding ribosome inhibitors or removing release factor 3 (RF3) also causes WT *Salmonella* to filament. Our work thus suggests that ribosome deficiency inhibits bacterial cell division via translational and transcriptional regulation of the histidine operon.

## RESULTS

### Slow ribosomes cause *Salmonella* filamentation within macrophages

**Fig 1:**
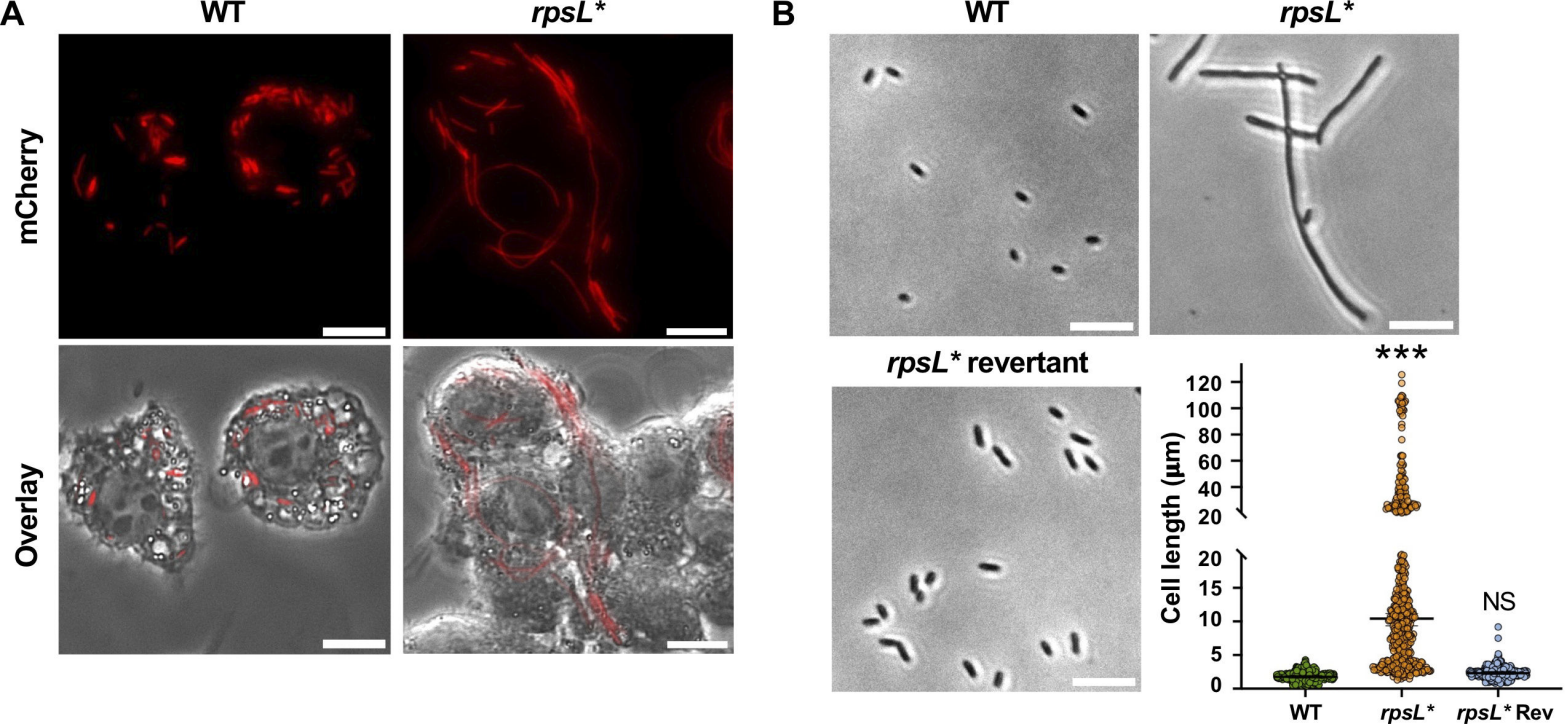
Filamentation of ribosome-defective *Salmonella* cells. (**A**) WT (14,028 s) and rpsL* Salmonella enterica Typhimurium cells carrying pZS P*_tet_*-mCherry were incubated with macrophages for 18 hours before imaging. Top, fluorescence microscopy. Bottom, overlay of fluorescence and phase-contrast images. Red cells indicate *Salmonella*. (**B**) *Salmonella* cells were grown in LPM, pH 4.5, for 16 hours before phase-contrast imaging. The dot plot shows quantitation of cell lengths in the *Salmonella* variants with over 400 cells for each strain from at least three biological replicates. The rpsL* revertant (Rev) has the chromosomal *rpsL* K42N mutation corrected to the WT. ***P < 0.001 (one-way ANOVA with Dunnett’s test). Scale bars: 10 µm.

*Salmonella* is an intracellular pathogen and can grow inside macrophages (38, 39). How translational regulation affects *Salmonella* growth inside host cells remains poorly understood. To test this, we first used a ribosomal mutant strain *rpsL* K42N (designated rpsL* in this study) that we previously generated (40). The *rpsL* gene encodes a conserved small subunit ribosomal protein uS12, which is located at the decoding center (41). Mutations in the *rpsL* gene confer Str resistance in many bacterial isolates, including *E. coli*, *Salmonella enterica*, *Mycobacterium tuberculosis*, and *Yersinia pestis* (32–36, 42). Earlier studies indicate that restrictive mutations in *rpsL* often result in decreased peptide elongation rates (37). Using a well-established LacZ assay (43, 44), we show that the rpsL* mutation in *Salmonella* indeed decreased the peptide elongation rate (Fig. S1). Next, we infected J774A.1 macrophages with WT, rpsL* Salmonella cells and allowed intracellular growth for 18 hours. Surprisingly, we found that whereas WT cells divided normally inside macrophages, rpsL* cells became filamentous, indicating a division defect during growth (Fig. 1A). Filamentation of rpsL* cells also occurred within RAW 264.7 macrophages (Fig. S2).

*Salmonella* cells grow in an acidic vacuole (pH < 5) inside macrophages (45). We thus used a low-phosphate low-magnesium (LPM) medium (46) with pH 4.5 to mimic the intracellular growth environment and recapitulated the filamentation phenotype of the rpsL* strain (Fig. 1B; Fig. S2). The median length of WT cells was about 2 µm in LPM, pH 4.5, while the median length of rpsL* cells was five times longer, with some growing to over 100 µm. To validate that the cell division defect is due to the rpsL* mutation, we reverted the K42N mutation on the chromosome and observed no filamentation in the revertant (rpsL* Rev in Fig. 1B). Similar to Salmonella rpsL*, E. coli rpsL* cells also exhibited filamentation in LPM, pH 4.5 (Fig. S3).

**Fig 2:**
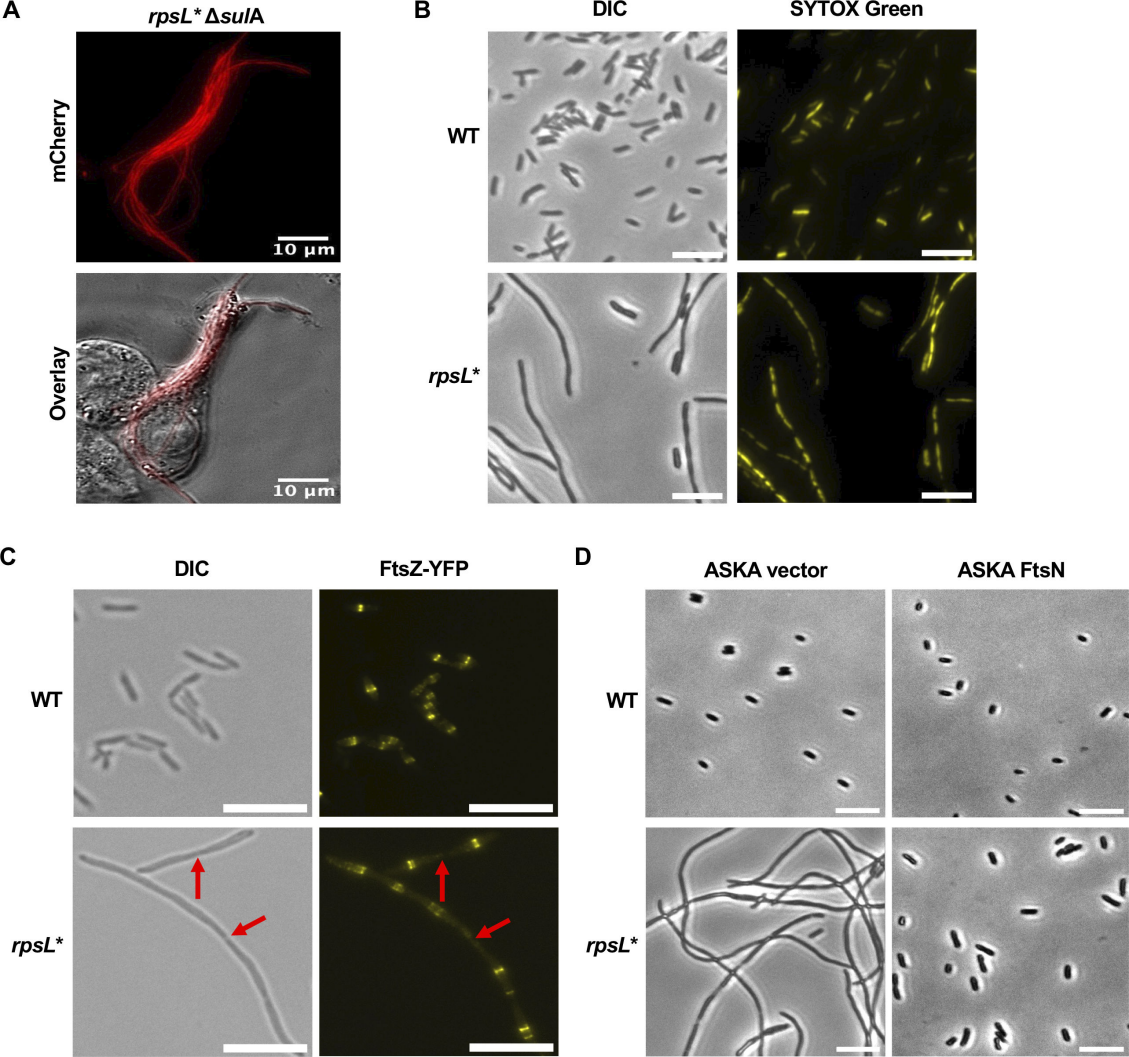
Rescue of rpsL* filamentation by FtsN overexpression. (**A**) Deleting *sulA* does not prevent rpsL* cells (red) from filamentation in macrophages. Macrophage infection and imaging are the same as Fig. 1A. (**B**) Differential interference contrast (DIC) and fluorescence microscopy of *Salmonella* cells treated with DNA marker SYTOX Green. The rpsL* filaments show multiple separated chromosomes. (**C**) DIC and fluorescence microscopy of *Salmonella* expressing FtsZ-YFP from the pZS plasmid. Multiple FtsZ rings are observed in filamentous cells. The red arrows indicate constriction sites that lack the FtsZ ring. (**D**) Phase-contrast images of WT and rpsL* Salmonella overexpressing late-divisome protein FtsN from the ASKA vector. Cells in panels **B–D** were grown in LPM, pH 4.5. All images are representative of at least three biological replicates. Scale bars: 10 µm.

### Filamentation of rpsL* Salmonella is caused by a late-division defect independent of SulA or PBP3_SAL_

Bacterial filamentation is often found to depend on SulA, an inhibitor of the key cell-division protein FtsZ induced by the SOS response (22, 47, 48). We found that deleting *sulA* did not prevent rpsL* cells from filamentation in macrophages (Fig. 2A). A previous report showed that *Salmonella* expresses a homolog of penicillin-binding protein 3 (PBP3) named PBP3_SAL_ under acidic conditions (49). Using a promoter reporter and qRT-PCR, we found that the level of PBP3_SAL_ transcription was not significantly reduced in the rpsL* background compared to WT (Fig. S4A and B). In addition, deleting the *PBP3_SAL_* gene in WT *Salmonella* did not lead to a filamentation phenotype (Fig. S4C). These results suggest that filamentation in rpsL* cells does not involve impaired expression of PBP3_SAL_.

To further characterize the division defect, we stained the chromosome with SYTOX Green and observed multiple segregated chromosomes in filamentous rpsL* cells, suggesting that such filamentation is not due to inhibition of DNA synthesis or chromosome separation (Fig. 2B). Bacterial cell division requires sequential assembly of the divisome (17). In *E. coli* and *Salmonella*, FtsZ first forms a ring at the septum and recruits late-division proteins. The FtsZ ring then disassembles before cell division completes (17). We examined the localization of FtsZ using a yellow fluorescent protein (YFP) fusion. Multiple FtsZ-YFP rings were observed in a typical filamentous cell (Fig. 2C). In addition, overexpression of a key late-division protein, FtsN, an important activator of division septum synthesis after the divisome has been assembled (50), abolished filamentation of rpsL* cells (Fig. 2D). Collectively, these results suggest that filamentation of rpsL* cells is caused by a defect in a later stage of cell division.

### Filamentation of rpsL* Salmonella requires an acid stress

**Fig 3:**
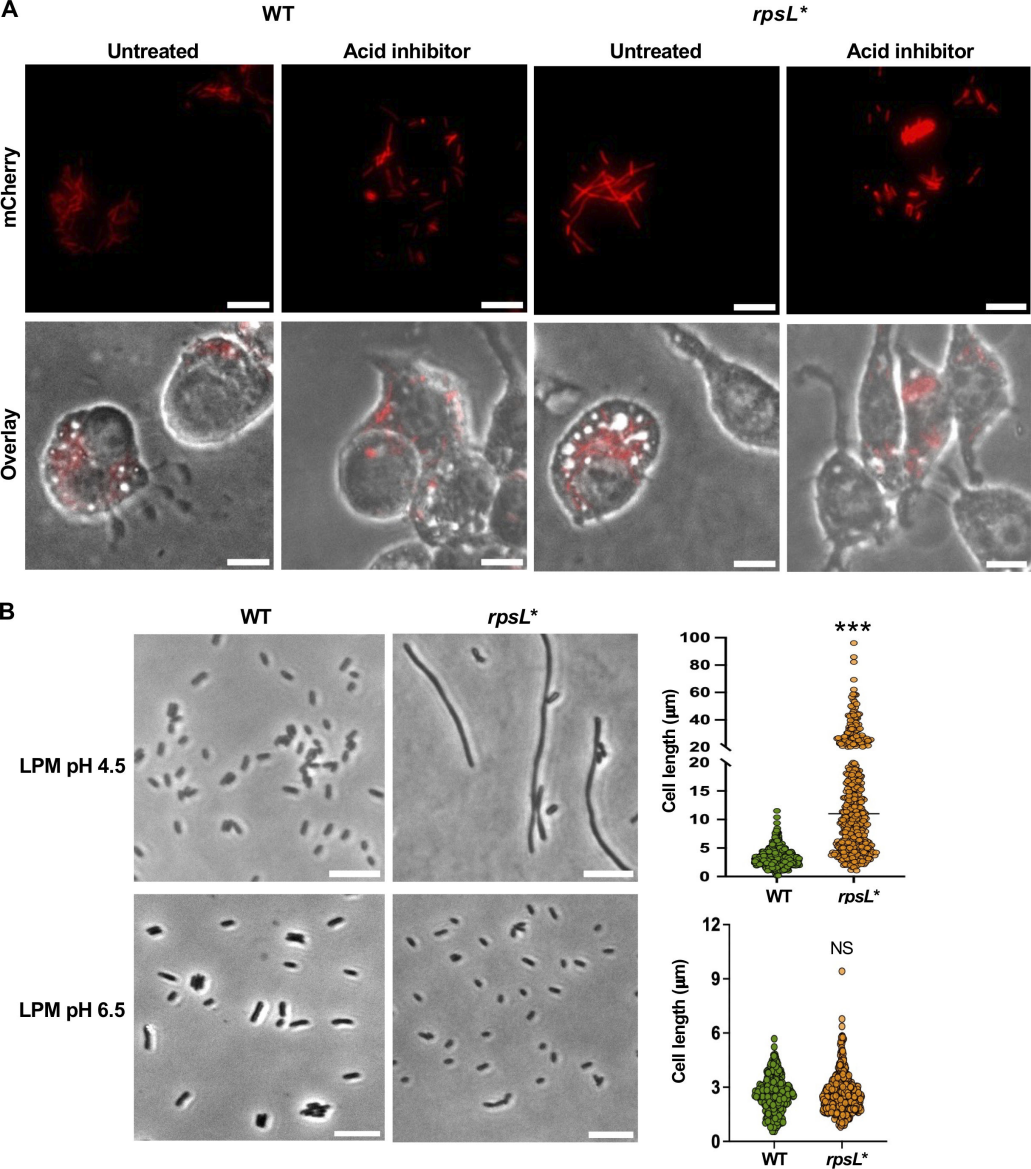
Filamentation of rpsL* cells under acidic pH. (**A**) WT and rpsL* Salmonella cells carrying pZS P*_tet_*-mCherry are incubated with macrophages for 18 hours with or without 100 nM CcA to suppress acid stress. (**B**) Phase-contrast imaging of *Salmonella* cells grown in LPM, pH 4.5, for 16 hours. The dot plots show the cell lengths of the *Salmonella* variants with over 200 cells for each strain from at least three biological replicates. ***P < 0.001; NS, not significant (unpaired *t*-test with Welch’s correction). All images are representatives of at least three biological replicates. Scale bars: 10 µm.

To test what host factors may induce *Salmonella* filamentation, we added 100 nM concanamycin A (CcA) to decrease acid stress during macrophage infection (51). CcA inhibits vacuolar ATPase and prevents acidification of the *Salmonella-*containing vacuole (51). The addition of CcA restores normal cell lengths for rpsL* cells grown in macrophages (Fig. 3A). In LPM media, increasing the pH to 6.5 also prevented rpsL* cells from filamenting (Fig. 3B). We next examined the morphologies of WT and rpsL* cells in other growth conditions. rpsL* cells were elongated in low phosphate, high magnesium medium at pH 4.5, and hyper-filamentous in high phosphate, low magnesium medium at pH 4.5 (Fig. S5). Removing amino acids from LPM at pH 4.5 did not prevent filamentation (Fig. S5). In the rich medium LB, lowering pH to 4.5 increased the cell length, but did not apparently induce filamentation. Collectively, these results indicate that acid stress is required for filamentation of rpsL* cells.

### Role of QueE in *Salmonella* filamentation

**Fig 4:**
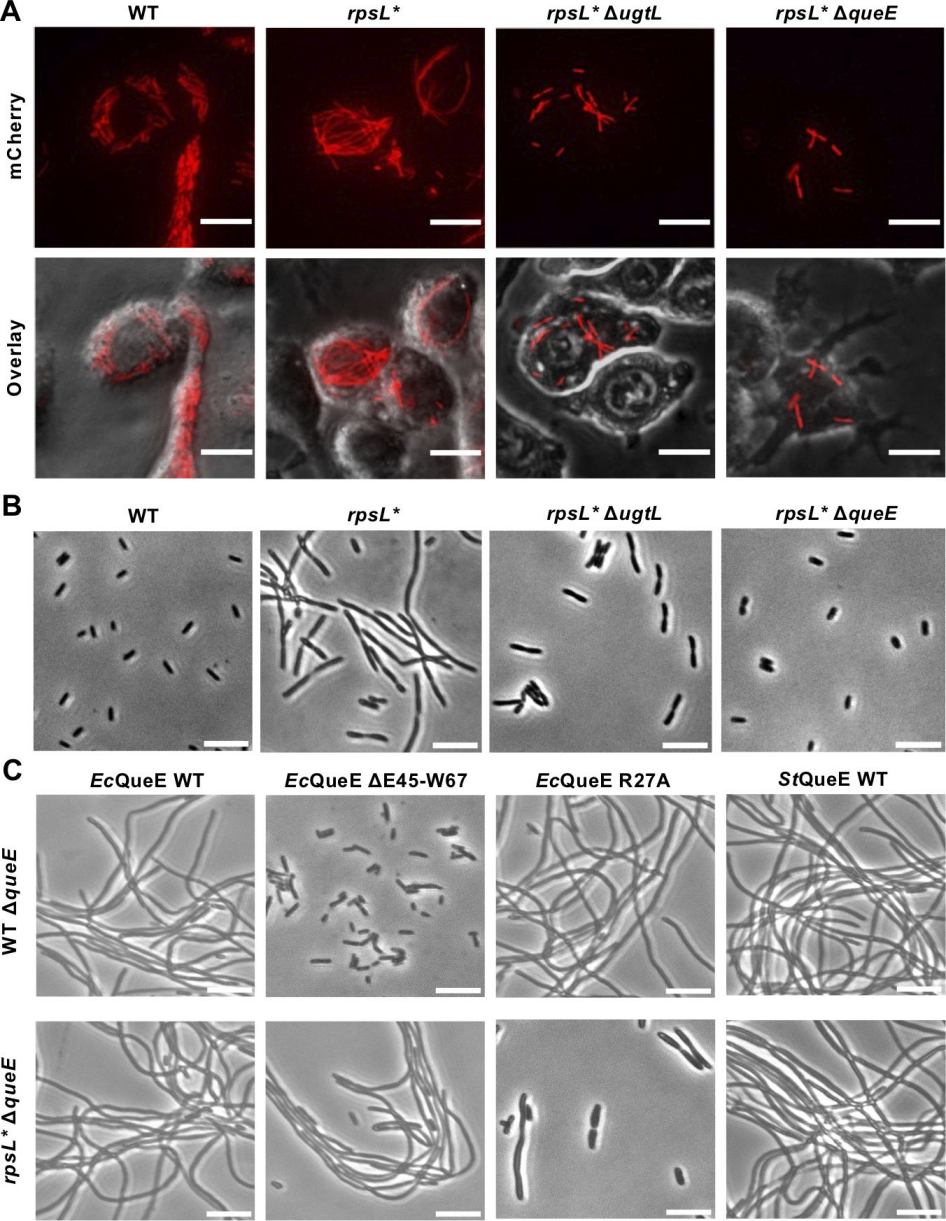
Effects of QueE on filamentation of WT and rpsL* Salmonella. (**A**) Macrophage infection by *Salmonella* variants in *rpsL*, *ugtL*, and *queE*; imaging as in Fig. 1A. (**B, C**) Phase-contrast imaging of *Salmonella* variants grown in LPM, pH 4.5, for 16 hours. *E. coli* (*Ec*) and *Salmonella* (*St*) QueE variants were overexpressed from plasmids. All images are representatives of at least three biological replicates. Scale bars: 10 µm.

Recent work has shown that *Salmonella* senses acid stress using UgtL, which activates PhoP phosphorylation (39, 52, 53). In *E. coli*, overactivation of PhoP by cationic antimicrobial peptides leads to inhibition of the divisome by QueE, which localizes at the septa when overexpressed (54, 55). We therefore deleted *ugtL* and *queE* in rpsL* Salmonella. The resulting rpsL* Δ*ugtL* and Δ*queE* cells no longer filamented in macrophages and in the acidic medium LPM, pH 4.5 (Fig. 4). To further test whether filamentation of rpsL* cells depends on QueE, we complemented the *queE* deletion strains with plasmid-borne *E. coli* and *Salmonella* QueE variants. In addition to inhibiting the divisome, QueE is also involved in tRNA queuosine modification (55). Deleting the region E45-W67 prevents QueE from inhibiting the divisome but does not affect tRNA queuosine modification, whereas the R27A mutation abolishes queuosine formation on tRNAs but does not affect divisome inhibition (55). We show that in WT Δ*queE Salmonella*, overexpressing *E. coli* or *Salmonella* WT QueE, but not ΔE45-W67 QueE, induces filamentation (Fig. 4C). This is consistent with the phenotype previously seen in *E. coli* (55). However, overexpressing ΔE45-W67 QueE in the rpsL* Δ*queE* strain does not abolish filamentation (Fig. 4C), suggesting that filamentation of rpsL* cells does not depend on the divisome-binding region of QueE. Further growth assays revealed that deleting *queE* in rpsL* (but not in WT *Salmonella*) inhibited cell growth in LPM, pH 4.5 (Fig. S6). Growth of rpsL* under acidic conditions appears to depend on the queuosine modification activity of QueE, as the R27A QueE mutant fails to restore growth in rpsL* (Fig. S6). We thus conclude that QueE is required for cell growth, rather than blocking cell division, in rpsL* Salmonella.

### Defective ribosomes activate the histidine operon to promote filamentation

**Fig 5:**
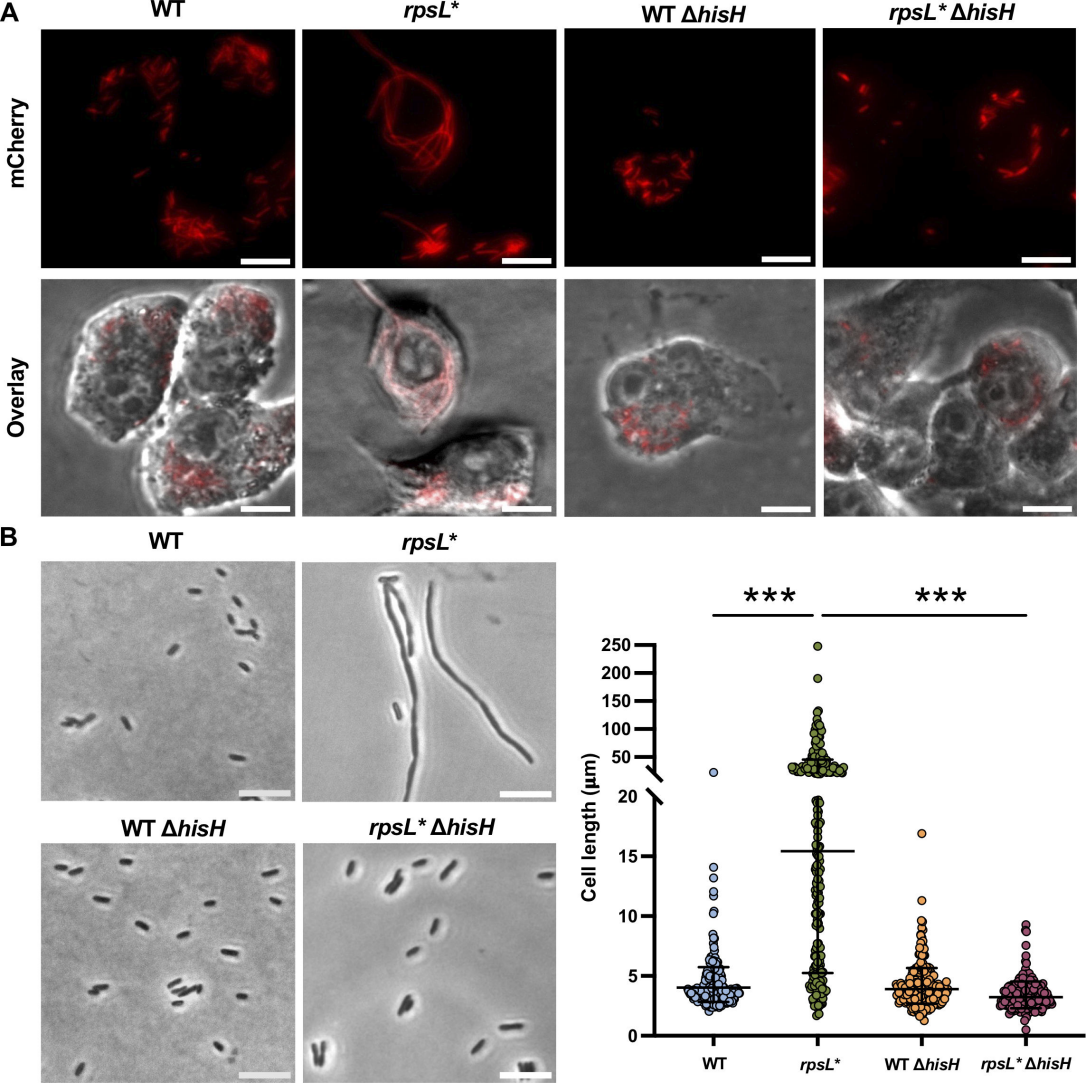
Effects of HisH on filamentation of rpsL* cells. (**A**) Macrophage infection by *Salmonella* variants. (**B**) Phase-contrast imaging of *Salmonella* variants grown in LPM, pH 4.5, for 16 hours. The dot plot shows quantitation of cell lengths in the *Salmonella* variants with over 200 cells for each strain from at least three biological replicates. ***P < 0.001; ns, not significant; one-way ANOVA with Dunnett’s test. All images are representatives of at least three biological replicates. Scale bars: 10 µm.

The acid stress in macrophages and *in vitro* is not sufficient to cause filamentation in WT *Salmonella* (Fig. 3), indicating that the rpsL* mutation further restricts cell division. Previous studies have revealed that filamentation of the SL1344 *Salmonella* strain in mammalian cells depends on a *hisG* mutation and that deleting *hisH* in the histidine operon (*hisGDCBHAFI*) abolishes filamentation (26). Overexpression of *hisHF* has also been linked to filamentation in *E. coli* and *Salmonella* (56, 57). Consistent with these studies, we found that deleting *hisH* in rpsL* Salmonella prevented filamentation in macrophages and *in vitro* (Fig. 5). In contrast to Δ*queE*, deleting *hisH* in rpsL* did not decrease growth in LPM, pH 4.5 (Fig. S7). HisH and HisF form the imidazole glycerol phosphate synthase complex, which is required for histidine biosynthesis. We show that overexpressing HisH alone, but not HisF, is sufficient to induce filamentation in WT *Salmonella* at acidic pH (Fig. S8), indicating that inhibition of cell division by HisH is independent of HisHF activity. Using mCherry-labeled HisH, we show that HisH is not enriched at division septa in filamentous rpsL* cells (Fig. S9), supporting the idea that HisH does not directly block the divisome.

**Fig 6:**
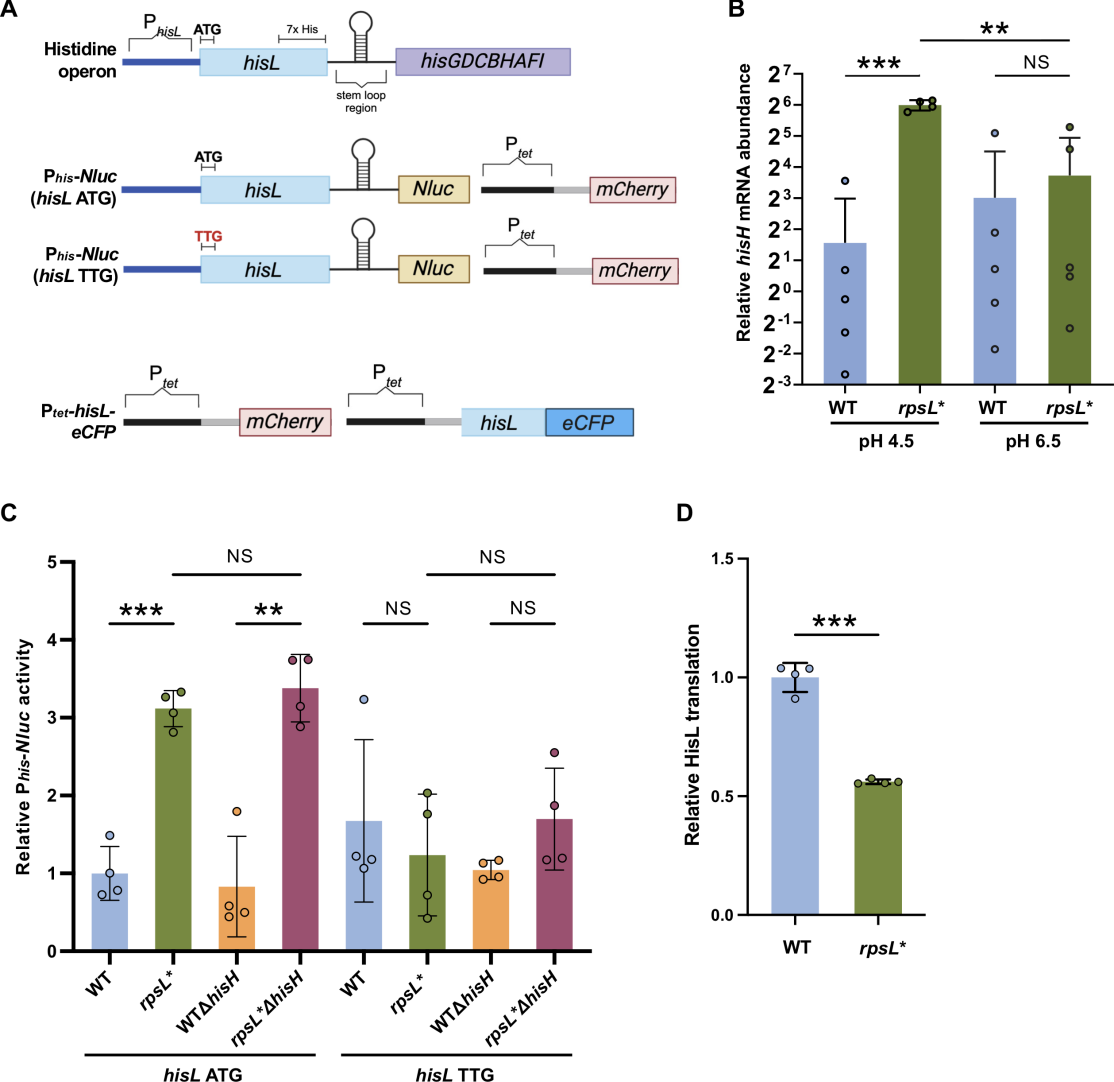
Translational regulation of *hisL* in *Salmonella*. (**A**) Scheme of the histidine operon and reporters. The *hisGDCBHAFI* operon is regulated by translation of the leader peptide HisL. The stem-loop region pauses RNA polymerase to attenuate transcription of the operon. HisL contains seven consecutive His codons. Upon His starvation, the ribosome pauses at HisL and promotes transcription of *hisGDCBHAFI*. The 5′ region of *hisG* is fused to the nano luciferase gene *Nluc* on the low-copy plasmid pZS, which also harbors *mCherry* under the control of a constitutive *tet* promoter. The ATG start codon of *hisL* is also mutated to TTG to abolish *hisL* translation. In the P*_tet_-hisL-eCFP* reporter, the *hisL* and *eCFP* coding regions are fused and controlled by P*_tet_*, and P*_tet_-mCherry* is used for normalization. (**B**) Relative *hisH* mRNA levels of *Salmonella* grown in LPM at pH 4.5 and pH 6.5 for 16 hours were measured by RT-qPCR. (**C**) Relative P*_hisG_-Nluc* activities of *Salmonella* grown in LPM, pH 4.5, for 16 hours normalized by mCherry. The rpsL* mutation increases *hisG* promoter activity, and mutating ATG to TTG of *hisL* abolishes the difference. (**D**) Relative HisL-eCFP translation calculated from the eCFP/mCherry ratio. Cells were grown in LPM, pH 4.5, for 16 hours. ***P < 0.001; NS, not significant; one-way ANOVA with Dunnett’s test (**B and C**); unpaired *t*-test with Welch’s correction (**D**).

Expression of the histidine operon is regulated by (p)ppGpp/DksA and translation of the HisL leader peptide (58–61) (Fig. 6A). Using reverse-transcription quantitative PCR (RT-qPCR), we found that the mRNA level of *hisH* was upregulated in rpsL* compared with WT cells when grown in LPM, pH 4.5, but not when the pH was increased to 6.5 (Fig. 6B). Removing DksA and the (p)ppGpp synthesis enzymes RelA and SpoT in rpsL* cells did not restore normal cell division (Fig. S10), suggesting that (p)ppGpp does not play a major role in filamentation of the rpsL* strain.

HisL is a small peptide with seven consecutive histidine codons near the C-terminus. During histidine starvation, the ribosome pauses at the histidine codons and promotes transcription of the downstream histidine operon, providing a feedback mechanism to increase histidine biosynthesis (58, 61). We fused the promoter region of *hisGDCBHAFI* to a nano luciferase gene (*Nluc*) and tested the expression of the resulting P*_hisG_-Nluc* (Fig. 6A). The *his* promoter (P*_his_*) activity was threefold higher in the rpsL* compared with the WT cells grown in LPM, pH 4.5 (Fig. 6C). However, the increased P*_his_* activity was not affected by the feedback loop, as shown by the *hisH* deletion mutants (Fig. 6C). Consistently, acidic northern blotting revealed that the aminoacylated tRNA^His^ levels were not statistically different between the WT and rpsL* strains (Fig. S11). These data suggest that the cellular histidine level is not a major determinant for rpsL* filamentation.

Mutating the ATG start codon of *hisL* to TTG decreased P*_his_* activity in rpsL* cells (Fig. 6C), demonstrating that upregulation of the histidine operon in the rpsL* depends on translation of *hisL*. In line with this, we show that translation of HisL-eCFP was less efficient in the rpsL* strain compared with the WT strain (Fig. 6D). These results indicate that the ribosome kinetic defect caused by the rpsL* mutation leads to slower translation of HisL and activation of the histidine operon.

### Ribosome inhibitors and termination defects induce filamentation

**Fig 7:**
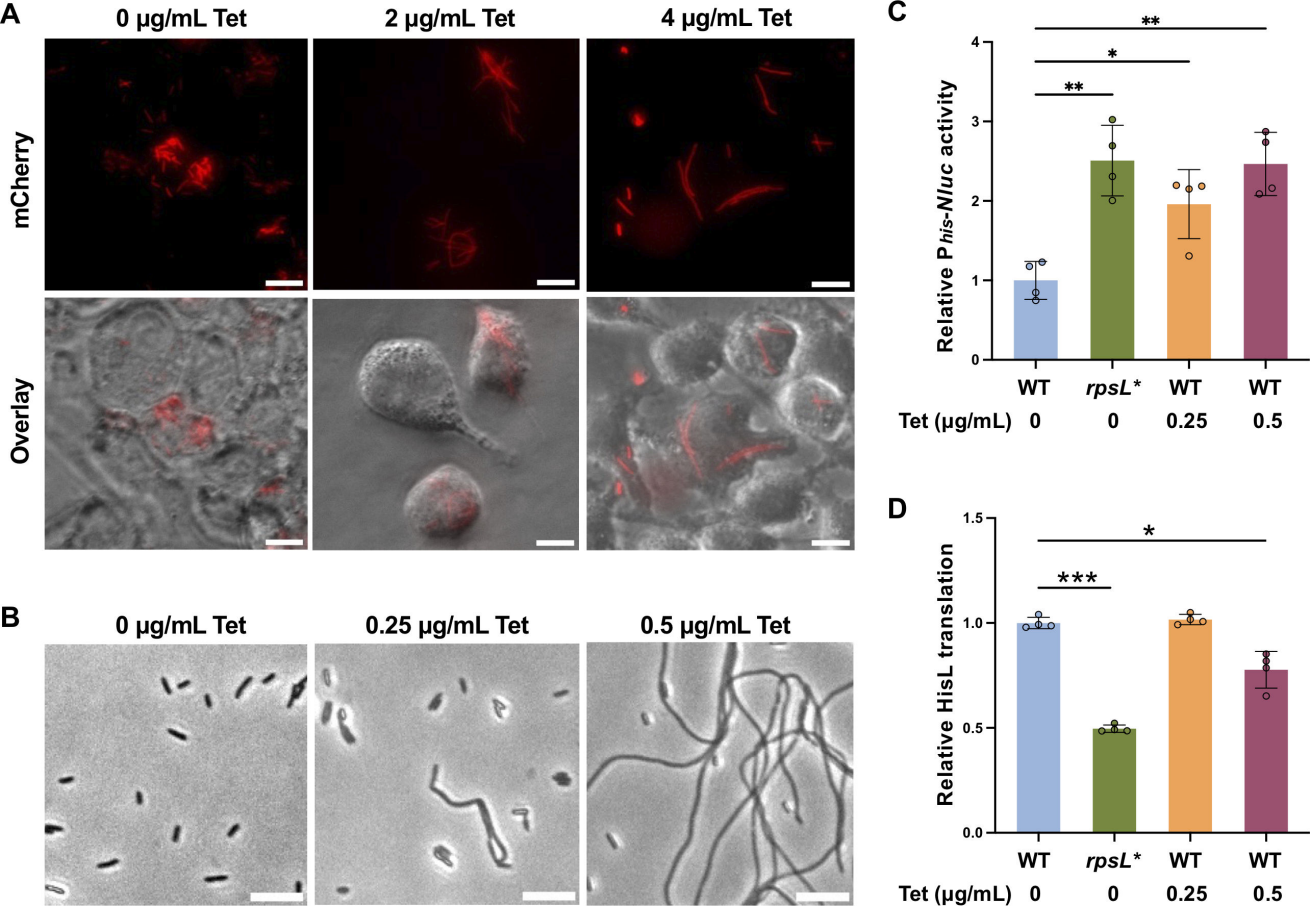
Effects of tetracycline on *Salmonella* filamentation. Growth of WT *Salmonella* (**A**) inside macrophages (18 hours) and (**B**) in LPM at pH 4.5 (16 hours) with or without Tet. (**C**) P*_hisG_* activities and (**D**) HisL-eCFP translation of WT *Salmonella* with or without Tet in LPM at pH 4.5. *P < 0.05; **P < 0.01; ***P < 0.001; one-way ANOVA with Dunnett’s test. All images are representatives of at least three biological replicates. Scale bars: 10 µm.

In addition to slowing translation, mutations in *rpsL* also increase ribosomal fidelity (32, 40, 42, 62). To further test whether ribosome deficiency impairs cell division independent of ribosomal fidelity, we treated WT *Salmonella* with ribosome-inhibiting antibiotics. The addition of tetracycline (Tet) and chloramphenicol (Chl) led to filamentous cells in WT *Salmonella* (Fig. 7; Fig. S12) at pH 4.5 but not pH 6.5. Like the rpsL* mutation, Tet treatment also increased the *his* promoter activity and decreased translation of HisL (Fig. 7C and D). In addition, filamentation was also induced in *Salmonella* and *E. coli* upon deletion of *prfC* (encoding RF3, Fig. S13), which can lead to ribosome pausing at the HisL stop codon (63). Our results collectively suggest that ribosome defects promote filamentation of *Salmonella* and *E. coli* under acidic conditions.

### Filamentation improves the survival of *Salmonella* under stress

**Fig 8:**
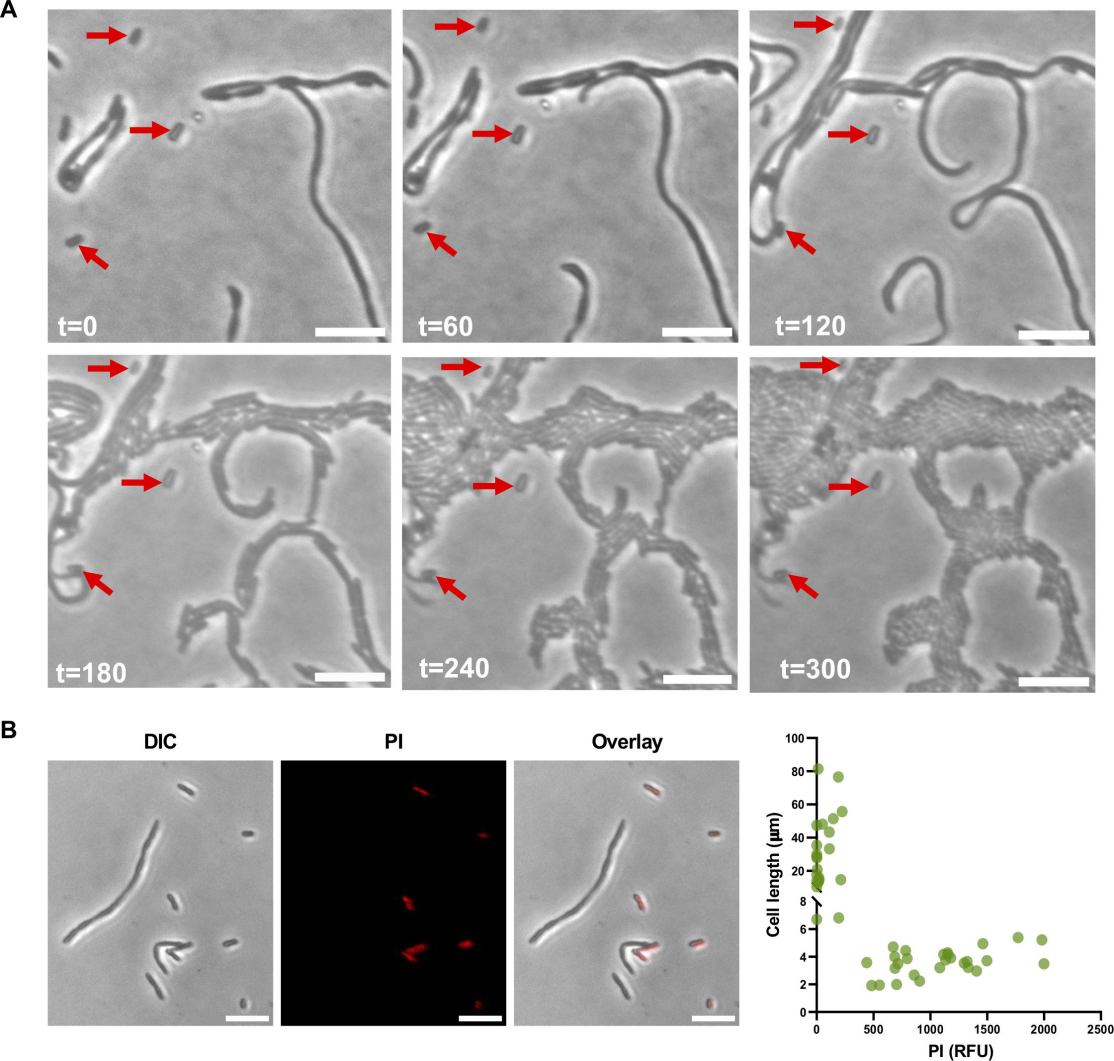
Survival of filamentous *Salmonella* cells under acid stress. (**A**) rpsL* Salmonella cells are grown in LPM, pH 4.5, for 16 hours and transferred to LB agarose pad for time-lapse microscopy. Filamentous cells resumed growth, whereas non-filamentous cells indicated by arrows mostly have stopped dividing. (**B**) rpsL* Salmonella cells are grown in LPM, pH 4.5, for 16 hours and stained with PI to indicate dead cells. The dot plot shows 50 cells from three biological replicates. RFU: relative fluorescence units. All images are representatives of at least three biological replicates. Scale bars: 10 µm.

Bacterial filamentation has been shown to serve as an adaptive strategy (19–22). To evaluate the fate of filamentous cells after stress, we performed time-lapse microscopy of rpsL* cells, which were grown in LPM, pH 4.5, overnight and moved to a Luria-Bertani (LB) agarose pad with a neutral pH. Most filamentous cells resumed cell division on LB, and the divisions occurred at multiple sites of the filament simultaneously (Fig. 8; Movie S1). Interestingly, short rpsL* cells failed to divide after the removal of acid stress. We next used propidium iodide (PI) to stain rpsL* cells grown overnight in LPM, pH 4.5, and found that, in contrast to the filamentous cells, short rpsL* cells were mostly killed under the acidic condition. Filamentation thus appears to facilitate rpsL* cells to survive acid stress.

## DISCUSSION

Bacterial pathogens undergo various stress conditions inside the host, such as nutrient starvation, antimicrobials, oxidative stress, and acid stress (39, 64–68). Many stress conditions have been shown to affect the efficiency and accuracy of protein synthesis (69, 70). In bacteria, amino acid starvation leads to the production of the alarmone (p)ppGpp, which attenuates ribosome biogenesis and translation initiation (13). Antibiotics and antimicrobial peptides frequently target the ribosome and inhibit protein synthesis (14, 15, 71, 72). Oxidative stress has also been shown to increase translational errors and decrease the peptide elongation rate (73, 74). Despite the mounting evidence that protein synthesis can be perturbed by genetic and environmental factors, our understanding of how the resulting translation stress affects bacteria-host interactions is still limited. A recent study shows that in *Salmonella enterica*, Mg^2+^ starvation (a condition that bacteria experience in host cells) attenuates protein synthesis via recruiting the chaperone DnaK to the ribosome (75). Such translation attenuation facilitates *Salmonella* survival during starvation (75). Previous work has shown that mutations in the ribosomal protein uS12 decrease colonization of *Salmonella* in mice and invasion of host cells (32, 40), but how ribosomal mutations affect the intracellular growth of bacteria remains unclear. In this study, we demonstrate that a restrictive mutation in uS12 (K42N) causes a cell division defect and filamentation in *Salmonella* cells grown inside macrophages and *in vitro* (Fig. 1; Fig. S2). Such filamentation depends on a low pH, because the addition of CcA to block the acidification of macrophages or increasing media pH abolishes filamentation of rpsL* cells (Fig. 3). Acid stress by itself does not induce filamentation of the WT *Salmonella*, suggesting that ribosome deficiency and acid stress act synergistically to block cell division. Previous work has shown that acidic pH decreases the activity of release factors *in vitro* and *in vivo*, leading to impaired termination of stop codons (76–78). It is possible that ribosome kinetic defects caused by the rpsL* mutation or ribosome inhibitors are amplified at acidic pH, resulting in severe ribosome stalling at the HisL leader peptide and enhanced expression of the histidine operon (see discussion below).

Previous work indicates that overexpressing the histidine operon causes *Salmonella* and *E. coli* to filament under certain conditions (56, 57). A *Salmonella* mutant strain overexpressing the histidine operon undergoes filamentation at 42°C but not at 37°C (56). Filamentation of a common lab strain of *Salmonella* Typhimurium SL1344 has also been observed in eukaryotic cells (25, 27, 28), which depends on a mutation in the *hisG* gene (*hisG46*) and the vacuolar environment (26). The *hisG46* mutation decreases histidine synthesis and likely activates the expression of the histidine operon. Deleting *hisH* or *hisF* prevents filamentation of SL1344 cells in macrophages. Interestingly, SL1344 cells grown *in vitro* divide normally, suggesting that host factors are also needed to promote filamentation (26). We show that deleting *hisH* abolishes filamentation of rpsL* Salmonella even under acidic conditions (Fig. 5). A recent transposon screening demonstrates that *hisF* is required for UPEC proliferation in the bladder cell infection cycle (79). It is intriguing to test whether the histidine operon also plays a role in UPEC filamentation. HisH and HisF form a dimer and are required for a late step in histidine synthesis. How HisHF affects cell division remains unclear, but it appears to be independent of its activities in histidine and purine biosynthesis (56, 57). In line with this notion, we show that overexpressing HisH alone is sufficient to induce filamentation in WT *Salmonella* (Fig. S8). HisH does not appear to localize at the septa and directly inhibit the divisome at late stages of cell division (Fig. S9), although we do not rule out transient recruitment of HisH to the early-stage divisome.

In *E. coli* and *Salmonella*, expression of the histidine operon is controlled by (p)ppGpp/DksA and the HisL leader peptide (58–61). Recent work shows that ppGpp and DksA regulate cell division in *E. coli* (80). However, filamentation of rpsL* cells does not appear to require ppGpp and DksA (Fig. S10). Our reporter assay shows that mutating the start codon of *hisL* abolishes the activation of the histidine operon in rpsL* (Fig. 6), suggesting that such regulation depends on *hisL* translation. As a trade-off for the high ribosome accuracy, *rpsL* mutations often decrease the translation rate (37). The rpsL* mutation does slow down translation elongation in *Salmonella* (Fig. S1), leading us to propose that translation deficiency of *hisL* synergizes with acid stress to impair *Salmonella* cell division and induce filamentation. This model is further supported by our results of ribosome-targeting antibiotics and the *prfC* deletion mutant (Fig. 7; Fig. S12 and S13).

Filamentation has been shown to facilitate bacterial survival under stress conditions and within hosts (21–23). For UPEC, filamentation in the bladder epithelial cells and urine plays a critical role in colonization and may help UPEC to disperse from host cells (21). Filamentation also helps the spread of the intracellular bacterium *Bordetella atropi* in animal cells (23). In addition, filamentation increases antibiotic tolerance and resistance by promoting DNA mutations (81), increasing signaling molecules (20), and reducing antibiotic uptake through decreasing the ratio of cell surface to volume (82). We have found that in overnight cultures of rpsL* cells grown at a low pH, filamentous cells are more viable than normal-sized cells (Fig. 8). It is therefore possible that *Salmonella* filamentation serves as a transient survival strategy under severe stress conditions within the host. Given that low concentrations of ribosome-targeting antibiotics promote filamentation, treatment with subinhibitory concentrations of these antibiotics may be counterproductive and prolong infections.

## MATERIALS AND METHODS

### Bacterial strains, plasmids, culture conditions, and reagents

All strains and plasmids used in this study are listed in Table S1. Gene deletion mutants were generated as previously described (83) using Chl as the selection marker, and the oligonucleotides used are listed in Table S2. Unless specified otherwise, all initial overnight cultures were grown in LB Lennox broth containing 10 g/L tryptone, 5 g/L sodium chloride, and 5 g/L yeast extract, at 37°C. When required, antibiotics were supplemented at final concentrations of 100 µg/mL ampicillin and 25 µg/mL Chl.

### *In vitro* filamentation assay

Overnight bacterial cultures were diluted 1:100 in LB Lennox broth and incubated at 37°C with shaking at 280 rpm until reaching an optical density at 600 nm (OD_600_) of approximately 0.4. Cells were harvested by centrifugation and transferred to a low phosphate, low magnesium (LPM, pH 4.5 or pH 6.5) medium and grown aerobically for 16 hours at 37°C with shaking. The composition of LPM medium was as previously described (46) and contained 5 mM KCl, 7.5 mM (NH_4_)_2_SO_4_, 0.5 mM K_2_SO_4_, 38 mM glycerol (0.3% vol/vol), 0.1% casamino acids, 8 µM MgCl_2_, 337 µM PO_4_^3-^, and 80 mM MES. The high magnesium medium contained 8 mM MgCl_2_, and the high phosphate medium contained 10 mM PO_4_^3-^.

### Microscopy of cells grown in media

Prior to imaging, bacterial cells grown in LPM media were pelleted by centrifugation to obtain a concentrated suspension with a final volume of approximately 20–50 µL. One microliter of the concentrated cell suspension was placed onto an agarose pad for immediate imaging. For fluorescence-based assays, bacterial cells were grown in LPM medium for 16 hours before being stained with either PI to identify dead cells or SYTOX Green to visualize nucleic acids. Fluorescence images were acquired using the same 60× objective lens and specific filters: the mCherry filter for PI and the YFP filter for SYTOX Green. Image analysis was carried out using the BZ-X800 Analyzer software (Keyence) or the ImageJ/Fiji software suite (National Institutes of Health). Bacterial cell lengths were quantified using the NeuronJ plugin in ImageJ/Fiji.

### Macrophage infection assays

Overnight cultures harboring the pZS P*_tet_-mCherry* plasmid were diluted 1:100 and grown in LB Miller medium (10 g/L tryptone, 10 g/L sodium chloride, and 5 g/L yeast extract) and incubated at 37°C with shaking for 5 hours to reach the early stationary phase. J774A.1 (ATCC TIB-67) or RAW264.7 macrophage cell lines were used for the infection with a multiplicity of infection (MOI) of 25. To synchronize the infection, plates were centrifuged at 1,000 × *g* for 5 minutes, followed by incubation at 37°C with 5% CO_2_ for 30 minutes. Subsequently, the infected cells were washed three times with phosphate-buffered saline (PBS) and supplemented with fresh DMEM containing 100 µg/mL gentamicin for 1 hour to kill extracellular bacteria. The monolayers were then maintained in DMEM containing 10 µg/mL gentamicin for an additional 16 hours.

### Bacterial RNA extraction and RT-qPCR

To prepare the samples for RT-qPCR analysis, overnight cultures were diluted 1:100 into fresh LB Lennox and incubated in a shaker at 280 rpm for 2 hours before being transferred to LPM medium. After 16 hours, the cell pellets were harvested by centrifugation and then flash frozen in liquid nitrogen and stored at −80°C until RNA extraction. Total RNA was extracted using the hot phenol method (84), and RNA samples were treated with RNase-free DNase (NEB) to remove genomic DNA contamination following the manufacturer’s protocol. Equal amounts of RNA from each sample were reverse transcribed into cDNA using the iScript cDNA Synthesis Kit (Bio-Rad). RT-qPCR was performed on 100 ng of cDNA using the One-Step RT-qPCR kit (BioLink). Primers used for the analysis are listed in Table S2. The 16S rRNA was used as an internal reference for normalization, and relative gene expression levels were calculated using the −∆∆Ct method.

### Luciferase assay

Overnight cultures were diluted 1:100 into fresh LB Lennox and incubated at 280 rpm for 2 hours, transferred to LPM media, and grown for an additional 16 hours. The cultures were then centrifuged to pellet the cells, washed once with PBS, and adjusted to an equivalent OD_600_ for all samples. A total of 100 µL from each culture was added to a 96-well white flat-bottom plate, and 1 µL of 5 mM furimazine stock solution was added to each well. Luminescence measurements were recorded at 10 minute intervals using a SYNERGY H1 microplate reader (BioTek). Luminescence values were normalized by constitutively expressed mCherry fluorescence, and blank PBS samples were used for background subtraction.

### Acidic northern blot

Total RNA was isolated from 4 mL of 16 hours of cultures grown in LPM media using the hot phenol method (84). RNA concentration was quantified, and samples were stored at −80°C until further analysis. For acidic gel electrophoresis, 2 µg of total RNA was loaded onto a 12% acidic urea-polyacrylamide gel (8 M urea) and electrophoresed overnight at 4°C using a sodium acetate running buffer (0.1 M sodium acetate, pH 4.5, 1 mM ethylenediaminetetraacetic acid). Following electrophoresis, RNA was transferred onto a Zeta-Probe membrane (Bio-Rad) and cross-linked using ultraviolet (UV) light. Hybridization was performed overnight at 42°C with 5′ end-labeled biotin oligonucleotide probes (see Table S2). tRNA^His^ signal was normalized to the signal of loading control 5S rRNA using ImageJ (NIH).

### Measurement of translation elongation rate

The translation elongation speed was measured using a method adapted from Zhu et al. (44). Briefly, bacterial cultures were grown at 37°C with shaking to mid-log phase in LB Lennox medium. To induce the expression of the *lacZ*α fusion gene, 5 mM IPTG was added to the cultures. At 10 second intervals, aliquots of 200 µL were collected into pre-chilled microtubes containing 10 µL of Chl (35 mg/mL) to stop further translation. Samples were immediately frozen in liquid nitrogen and stored at −80°C until analysis. Prior to LacZ activity measurement, cell samples were incubated at 37°C in a water bath for 1 hour to ensure that the newly synthesized LacZα fragment fully complemented the LacZω fragment. The LacZ activity was assessed using the fluorescence substrate 4-methylumbelliferyl-D-galactopyranoside (MUG). The reactions were stopped by 1 M Na_2_CO_3_. Fluorescence intensity was measured using a SYNERGY H1 microplate reader (BioTek) with a 365 nm excitation filter and a 450 nm emission filter.

### Measurement of bacterial growth

Overnight cultures were diluted 1:50 and grown in LPM, pH 4.5, at 37°C with vigorous shaking. The optical density at 600 nm (OD_600_) was measured every 20 minutes using a SYNERGY HTX microplate reader (BioTek).

### Time-lapse microscopy

Bacterial cultures were grown in LPM medium for 16 hours and pelleted by centrifugation to obtain a concentrated suspension. Two microliters of the concentrated culture was placed on a 1.5% agarose LB pad within a Gene Frame (Thermo Fisher Scientific). Images were captured at 20 minute intervals using a BZ-X800 fluorescence microscope (Keyence) equipped with a 60× phase-contrast objective lens. Image analysis was performed using the BZ-X800 analyzer software (Keyence).

### Statistical analyses

All images presented are representative of at least three biological replicates. Statistical significance was evaluated using one-way analysis of variance (ANOVA) followed by Dunnett’s post-hoc test or unpaired *t*-tests with Welch’s correction, depending on the experimental design. Statistical thresholds are defined as follows: *P* values less than 0.05 are considered significant (*), while *P* values below 0.01 and 0.001 are regarded as highly significant (** and ***, respectively).

## Data Availability

All study data discussed in the paper are available in the main text and supplemental material.

## SUPPLEMENTAL MATERIAL

Supplemental Figures and Tables mbio.01417-25-s0001.pdf Figures S1-S13, Tables S1 and S2, Movie S1 legend, and supplemental references.

Movie S1 mbio.01417-25-s0002.avi Regrowth of filamentous *rpsL* Salmonella* after acid stress removal.

ASM does not own the copyrights to Supplemental Material that may be linked to, or accessed through, an article. The authors have granted ASM a non-exclusive, world-wide license to publish the Supplemental Material files. Please contact the corresponding author directly for reuse.
