# Supplementary material for: Ribosome deficiency induces Salmonella filamentation within host cells
Source: mBio. 2025 Jun 30;16(8):e01417-25. doi: 10.1128/mbio.01417-25 (PMC12345169; doi:10.1128/mbio.01417-25)
Supplement: Supplemental Figures and Tables — Figures S1-S13, Tables S1 and S2, Movie S1 legend, and supplemental references. [file mbio.01417-25-s0001.pdf]

Ribosome Deficiency Induces *Salmonella* Filamentation within Host Cells

Zhihui Lyu[1†], Cierra Wilson[1†], Kalyn Weiss[2], Spencer Lewis[1], Kurt Fredrick[3], William Margolin[2#], Jiqiang Ling[1#]

[1]Department of Cell Biology and Molecular Genetics, The University of Maryland, College Park, MD 20742, USA

[2]Department of Microbiology and Molecular Genetics, McGovern Medical School, The University of Texas Health Science Center, Houston, TX 77030, USA

[3]Department of Microbiology, The Ohio State University, Columbus, OH 43210, USA

[†]The first two authors contributed equally to this work. The order of the co-first authors was determined alphabetically based on the last names.

[#]Correspondence: Jiqiang Ling (Tel: +1 3014051035. Email: jling12@umd.edu) or William Margolin (Tel: +1 7135005452. Email: William.Margolin@uth.tmc.edu)

Running title: Translational Control of Bacterial Cell Division

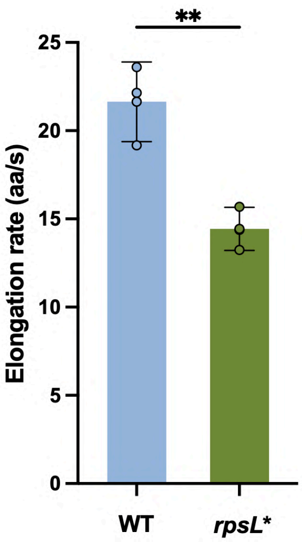

Figure S1. Translation elongation rates of *Salmonella* strains. The elongation rates were measured using a *lacZ* reporter as described (1). aa: amino acids. ** P < 0.01 (unpaired t-test).

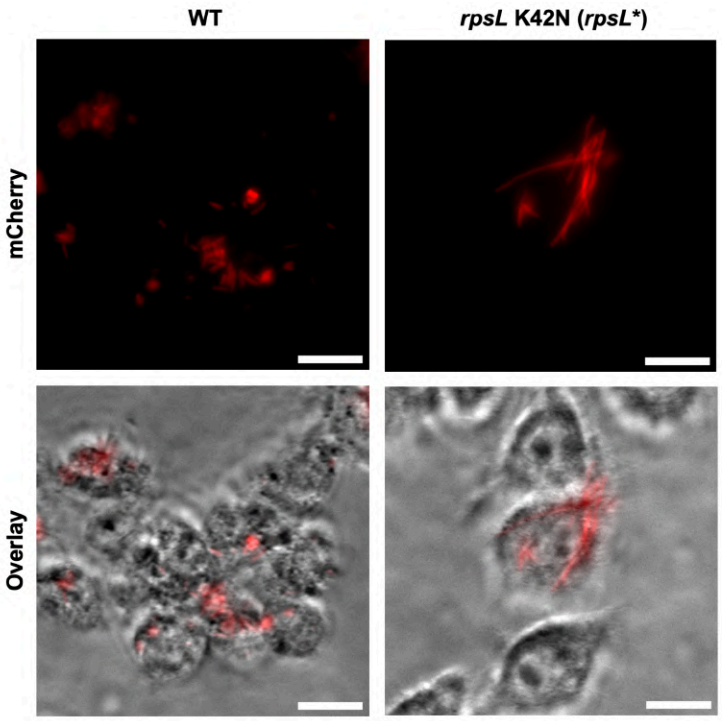

Figure S2. *Salmonella* filamentation in RAW 264.7 macrophages. Growth of *Salmonella* variants in RAW 264.7 macrophages for 18 hours. The images are representatives of three biological replicates. Scale bars: 10 μm.

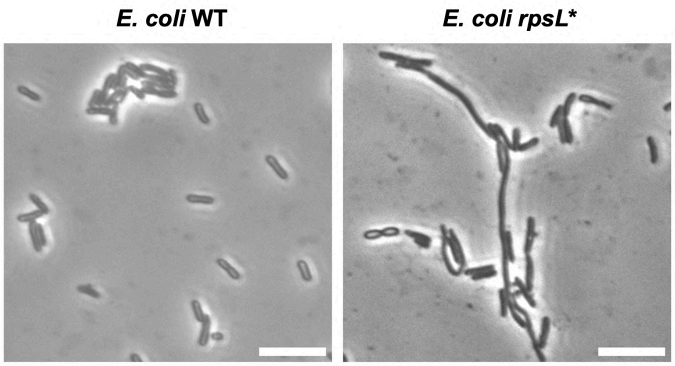

Figure S3. Filamentation of *rpsL* E. coli* under acidic conditions. WT *E. coli* (MG1655) and *rpsL** cells were grown in LPM pH 4.5 for 16 hours before phase-contrast imaging. The images are representatives of three biological replicates. Scale bars: 10 μm.

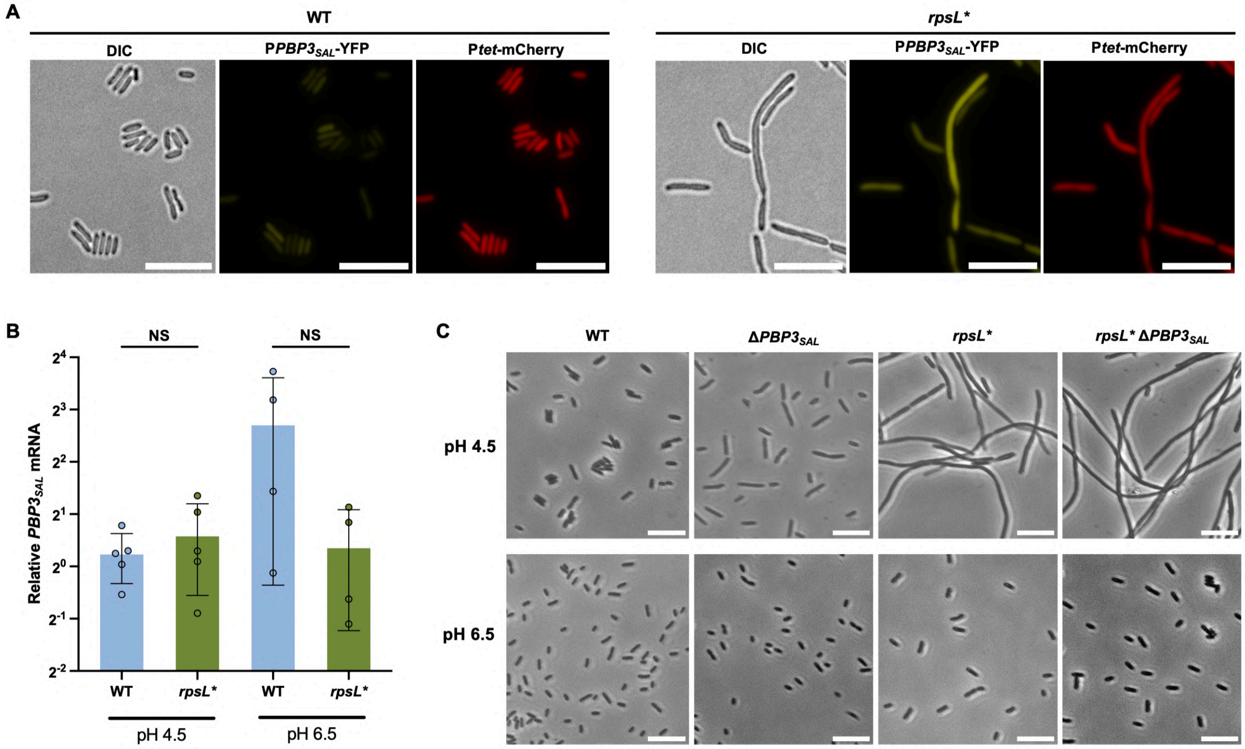

Figure S4. Effects of PBP3$_{SAL}$ on *Salmonella* filamentation. (A) Expression of *PBP3$_{SAL}$*-YFP in WT and *rpsL* Salmonella* cells grown in LPM pH 4.5. P*tet*-mCherry was used as a constitutive promoter control. The same exposure time was used for the WT and *rpsL** YFP images. Filamentous cells did not exhibit a lower *PBP3$_{SAL}$* promoter activity. (B) Relative mRNA level of *PBP3$_{SAL}$* in *Salmonella* cells grown in LPM pH 4.5 or 6.5 determined by qRT-PCR. NS, not significant based on one-way ANOVA with Dunnett's test. (C) Phase-contrast images of *Salmonella* variants grown in LPM pH 4.5 or 6.5. All images are representatives of at least three biological replicates. Scale bars: 10 μm.

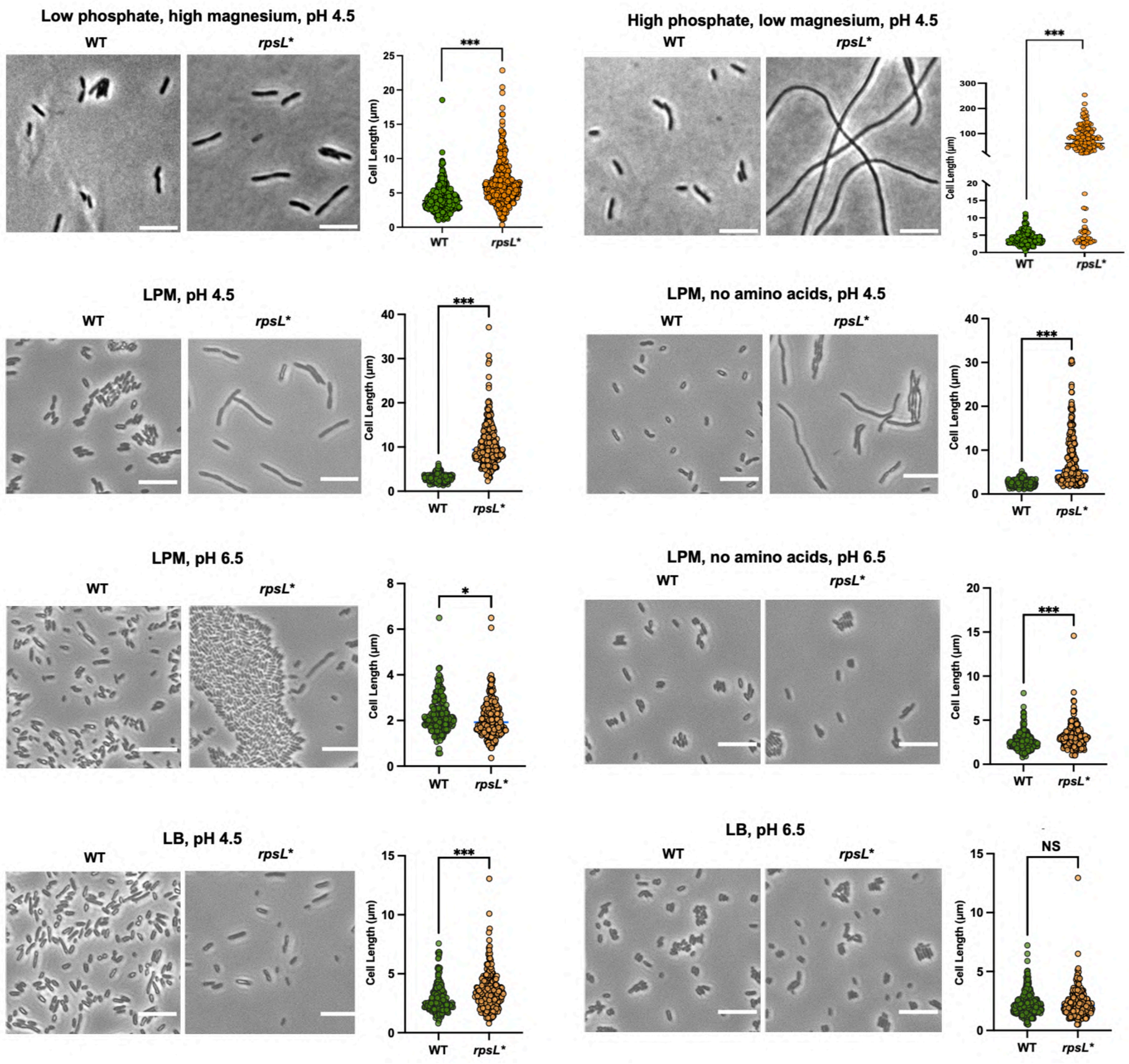

Figure S5. Effects of medium composition on *Salmonella* filamentation. Phase-contrast imaging of *Salmonella* cells grown in various media for 16 h. All images are representatives of at least two biological replicates. LPM, low phosphate (337 μM), low magnesium (8 μM); high phosphate (10 mM); high magnesium (8 mM); LB, Luria-Bertani. * $P < 0.05$, *** $P < 0.001$; NS, not significant (unpaired t-test with Welch's correction). Scale bars: 10 μm.

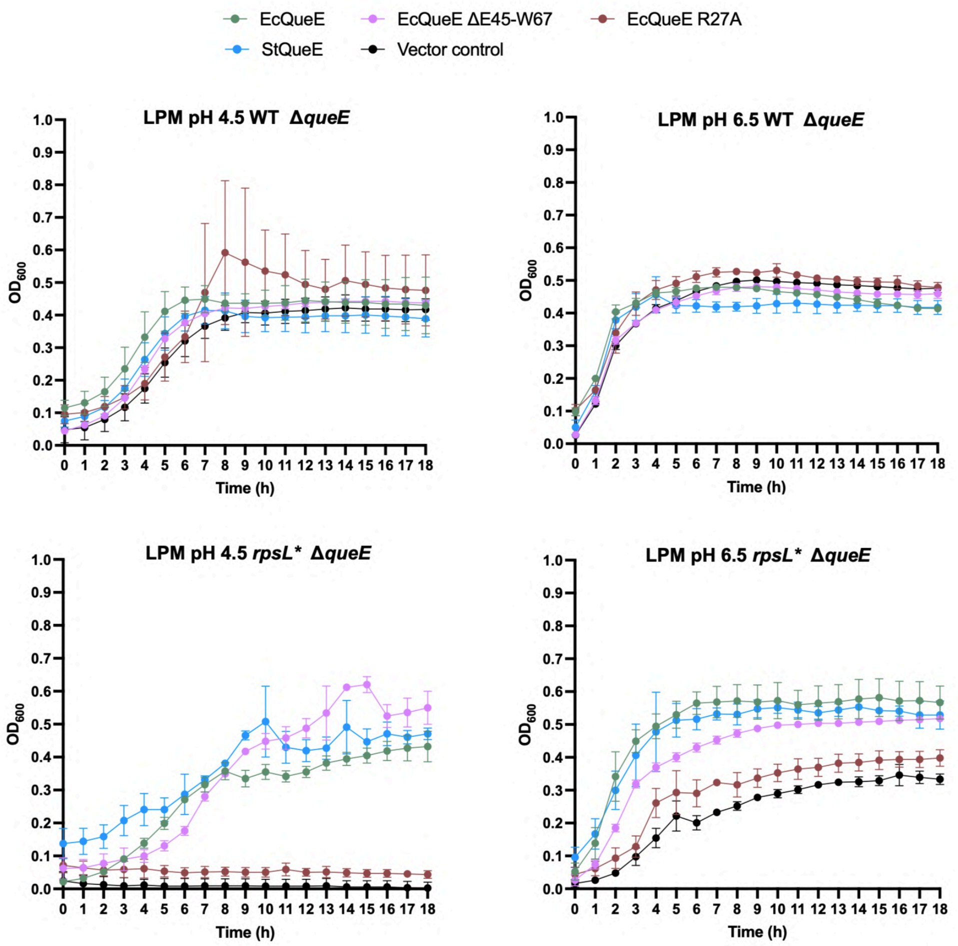

Figure S6. Growth of QueE complemented *Salmonella* strains. WT and *rpsL** strains lacking native *queE* were complemented with QueE variants from *E. coli* or *S.* Typhimurium as in Figure 4C. The error bars represent the standard deviation of three biological replicates.

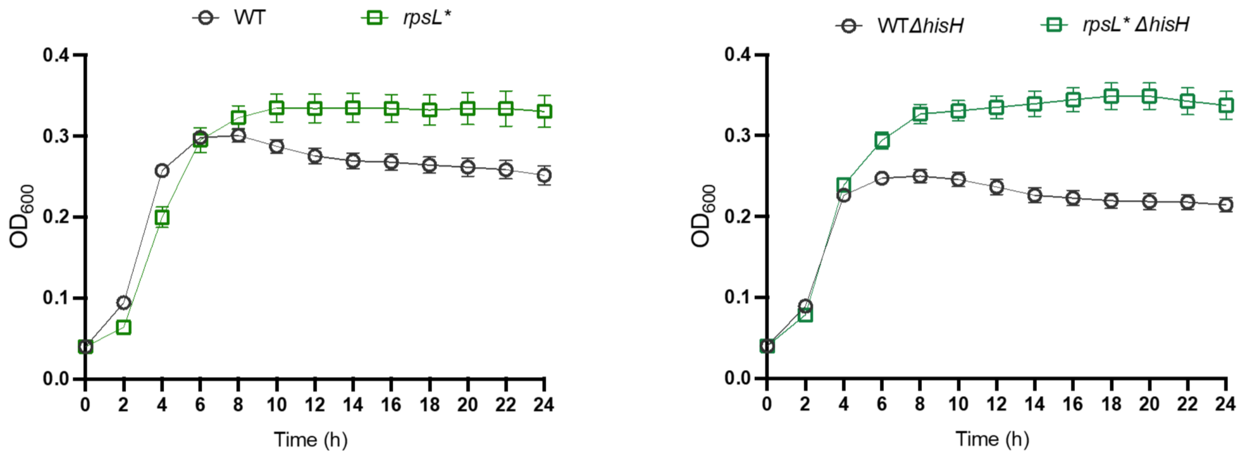

Figure S7. Growth of *Salmonella* variants in LPM pH 4.5. The error bars represent the standard deviation of three biological replicates.

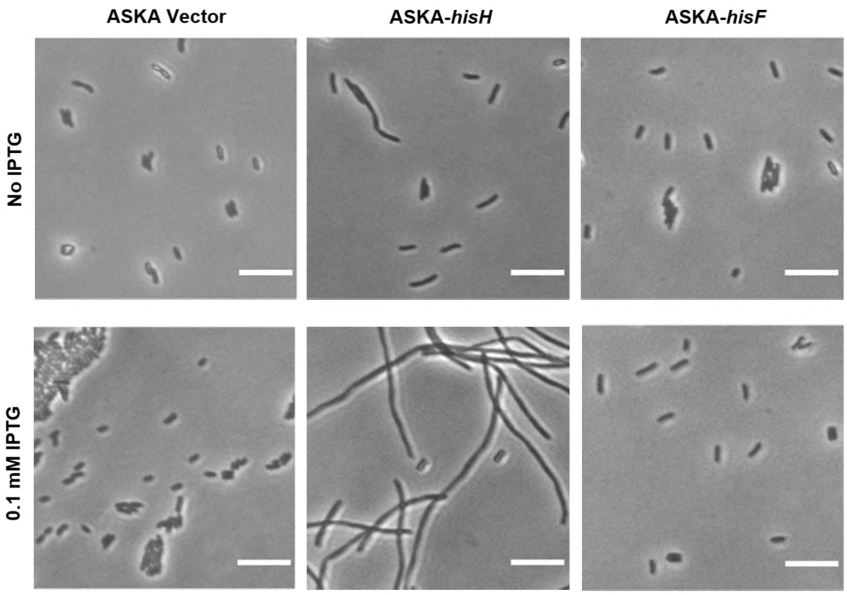

Figure S8. Overexpression of HisH in WT *Salmonella.* Expression of *E. coli* HisH or HisF from the ASKA vector was induced by the addition of IPTG. The phase contrast images are representatives of three biological replicates. Scale bars: 10 µm.

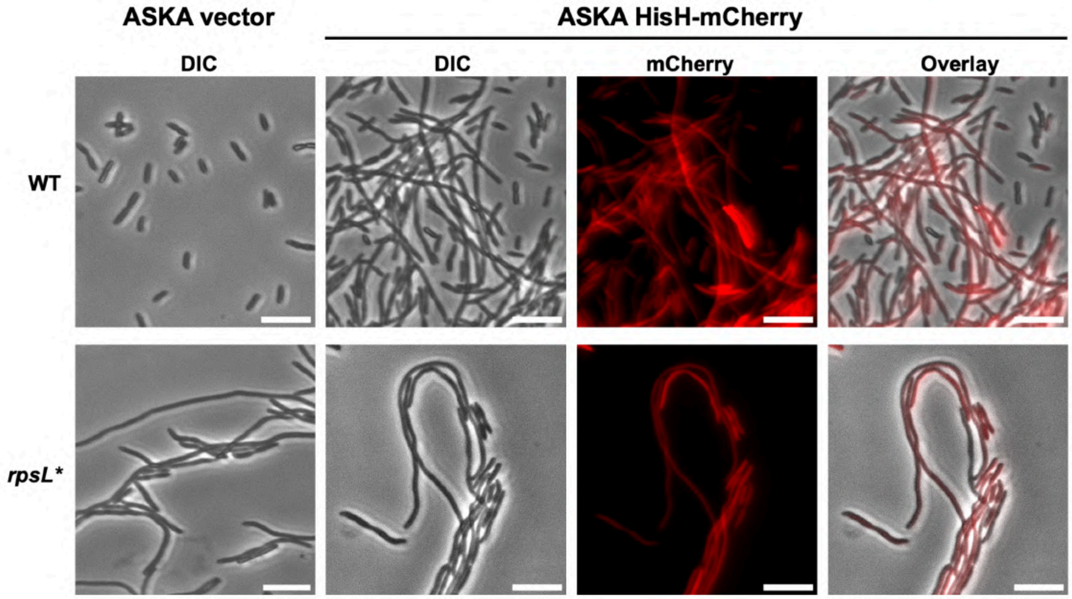

Figure S9. Overexpression of HisH-mCherry in *Salmonella* cells. *Salmonella* HisH-mCherry was expressed from the ASKA vector in WT and *rpsL** cells grown in LPM pH 4.5. Overexpressing HisH-mCherry is sufficient to induce filamentation of WT cells, and His-mCherry was localized diffusely in the cytoplasm. The images (phase contrast, mCherry fluorescence) are representatives of three biological replicates. Scale bars: 10 μm.

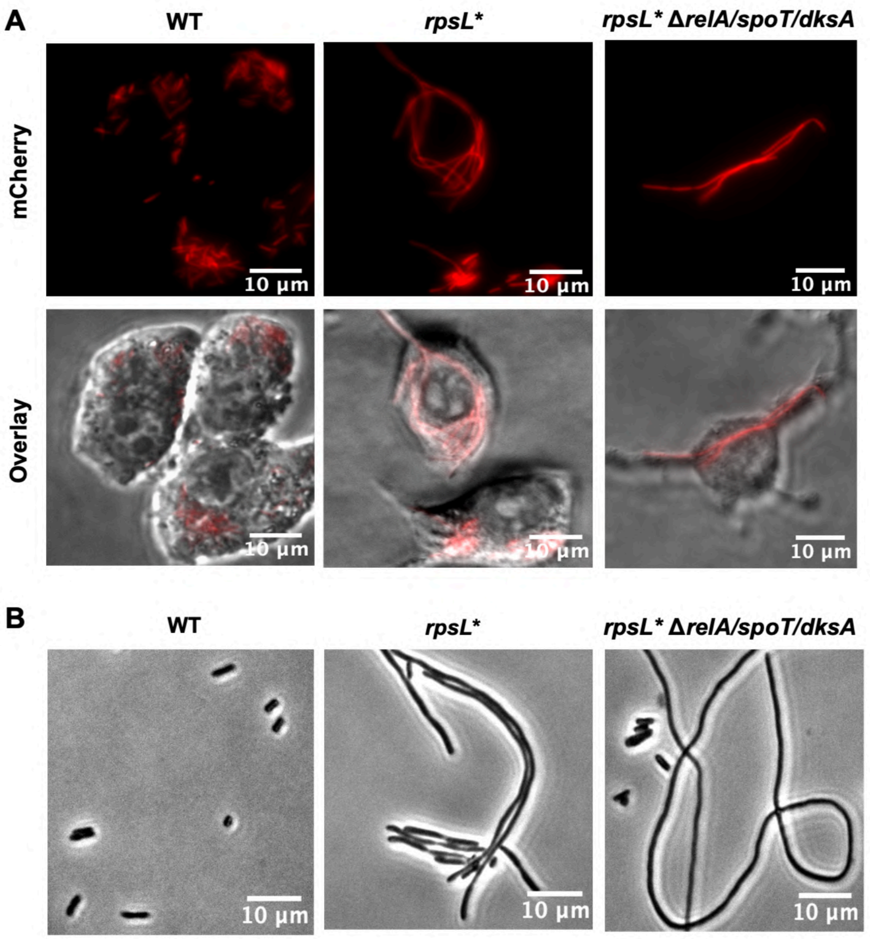

Figure S10. Effects of (p)ppGpp on filamentation of *rpsL cells.** (A) Macrophage infection by *Salmonella* variants for 18 hours. (B) Phase-contrast and fluorescence imaging of *Salmonella* variants grown in LPM pH 4.5 for 16 hours. All images are representatives of at least three biological replicates.

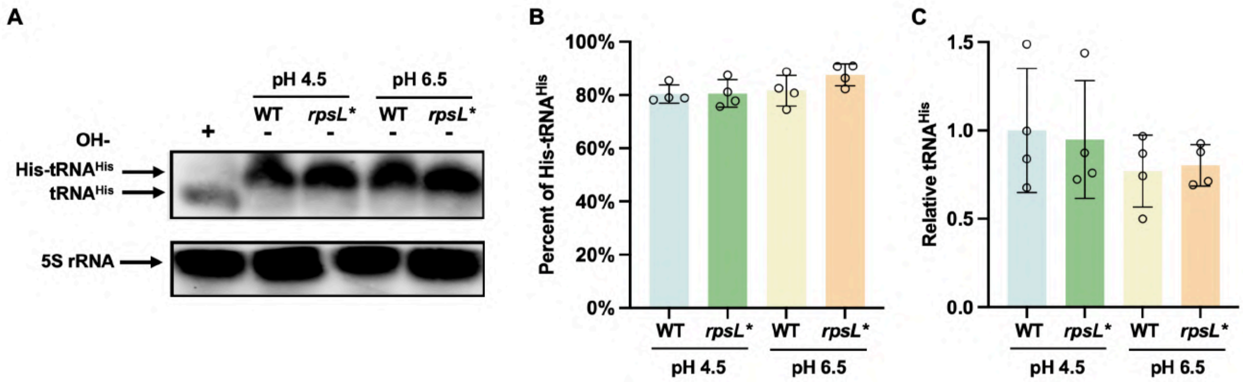

Figure S11. Aminoacylation levels of $tRNA^{His}$ in WT and *rpsL.** (A) Acidic northern blot of $tRNA^{His}$ from WT and *rpsL** *Salmonella* grown in LPM pH 4.5 for 16 hours. Alkaline (OH^-) treatment removes amino acids from $tRNA^{His}$. (B) and (C) show quantitation of the band intensities. There are no statistically significant differences between WT and *rpsL** analyzed by one-way ANOVA with Dunnett's test.

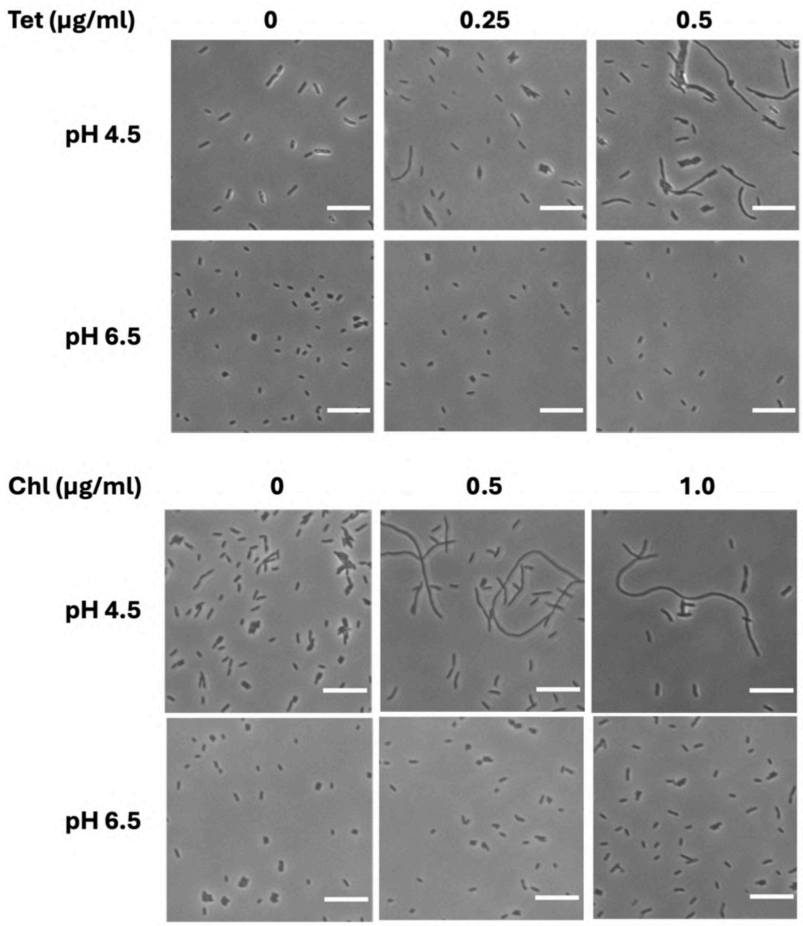

Figure S12. Effects of ribosome inhibitors on filamentation in WT *Salmonella*. Cells were grown in LPM pH 4.5 and 6.5 with or without tetracycline (Tet) or chloramphenicol (Chl) for 16 hours before phase-contrast imaging. All images are representatives of at least three biological replicates. Scale bars: 10 μm.

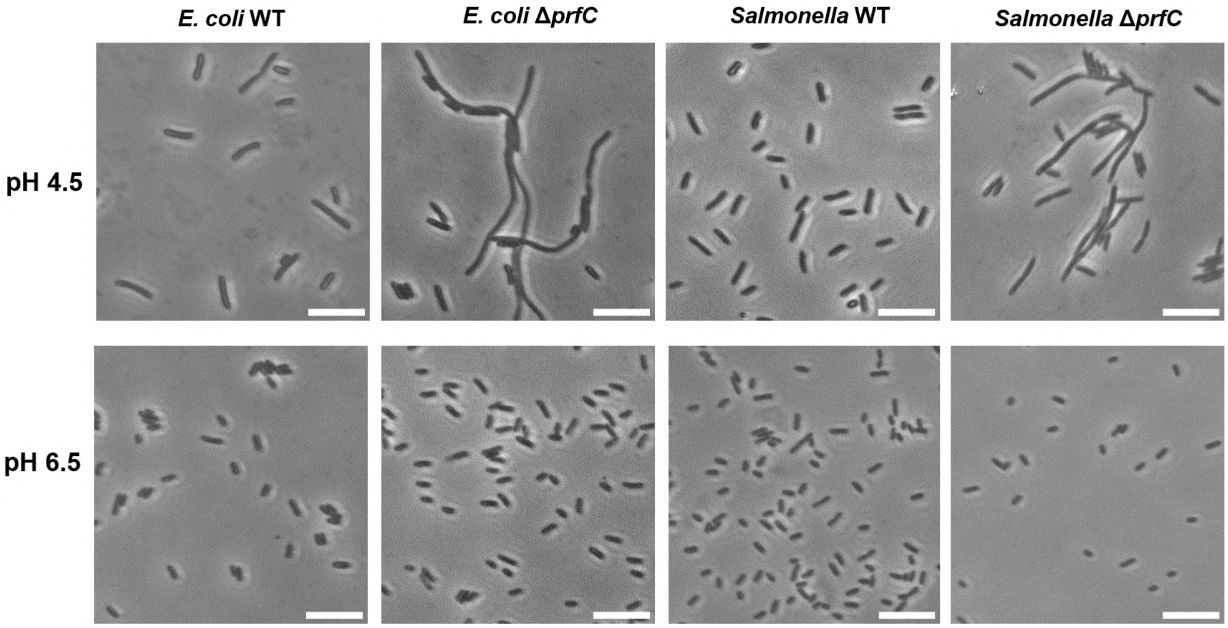

Figure S13. Effects of release factor on cell division. Cells were grown in LPM pH 4.5 and 6.5 for 16 hours before phase-contrast imaging. All images are representatives of at least three biological replicates. Scale bars: 10 μm.

Table S1. Strains and plasmids used in this study.

Strains	Description	Source
S. Typhimurium ATCC 14028s	WT	ATCC
S-*rpsD**	*Salmonella rpsD* I199N	Lab collection
S-*rpsL**	*Salmonella rpsL* K42N	Lab collection
S-*rpsL* K42T	*Salmonella rpsL* K42T	This study
WT Δ*phoP*	WT *ΔphoP*::Chl	Lab collection
*rpsL** Δ*phoP*	*rpsL** Δ*phoP*::Chl	Lab collection
WT Δ*queE*	WT Δ*queE*::Chl	This study
*rpsL** Δ*queE*	*rpsL** Δ*phoP*::Chl	This study
WT Δ*ugtL*	WT *ΔugtL*::Chl	This study
*rpsL** Δ*ugtL*	*rpsL** Δ*ugtL*::Chl	This study
WT Δ*hisH*	WT Δ*hisH*::Chl	This study
*rpsL** Δ*hisH*	*rpsL* ΔhisH*::Chl	This study
WT Δ$PBP3_{SAL}$	WT Δ$PBP3_{SAL}$::Chl	This study
*rpsL** Δ$PBP3_{SAL}$	*rpsL** Δ$PBP3_{SAL}$::Chl	This study
Δ*relA/spoT/dksA*	*Salmonella* Δ*rel*::FRT/*spoT*::FRT/*dksA*::Chl	This study
WT Δ*prfC*	WT Δ*prfC*::Chl	This study
*rpsL** Δ*sulA*	*rpsL** Δ*sulA*::Chl	This study
MG1655	*E. coli* K-12 MG1655 (F-, λ⁻, rph-1)	Lab collection
MG *rpsL**	MG *rpsL* K42N	Lab collection
MG Δ*prfC*	MG Δ*prfC*::Chl	Lab collection

Plasmids	Description	Source
pZS-P*tet-mCherry*	Rep101; Amp^r	Lab collection
pZS-P*tet-lacZ*	Rep101; Amp^r	Lab collection
pZS-P*araBAD*-FtsZ-YFP	Rep101; Amp^r	This study
pKD46	Rep101; Amp^r	(2)
pKD3	R6K γ ori; Amp^r and Cam^r	(2)
pCP20	Rep101(Ts); Amp^r and Cam^r	(2)
ASKA vector	ASKA *E. coli* open reading frame library; Cam^r	(2)
ASKA-FtsN	ASKA *E. coli* open reading frame library; Cam^r	(2)
ASKA-HisH	ASKA *E. coli* open reading frame library; Cam^r	(2)
ASKA-HisF	ASKA *E. coli* open reading frame library; Cam^r	(2)

pQueE-WT (pRL03)	Overexpression of WT *E. coli* QueE; Amp^r	(3)
pQueE-ΔE45-W67 (pSA62)	Overexpression of ΔE45-W67 *E. coli* QueE; derived from pRL03	(3)
pQueE-R27A (pSY98)	Overexpression of R27A *E. coli* QueE; derived from pRL03	(3)
pSTQueE (pSA21)	Overexpression of WT *Salmonella* QueE; derived from pRL03	(3)
pZS- P*tet*-*mCherry*-P*PBP3*$_{Sal}$-*YFP*	Rep101; Amp^r	This study
pZS- P*tet*-*mCherry*-P*hisL*-*Nluc*	Rep101; Amp^r	This study
pZS- P*tet*-*mCherry*-P*hisL*-TTG-*Nluc*	Rep101; Amp^r	This study
pAG306-pTet07-*Nluc*	pMB1 ori; Amp^r	(4)

Table S2. Oligonucleotides used in this study.

Oligonucleotide name	Oligonucleotide sequence (5'-3')	Description
queE-P1	cgaaatctgcaataattaacgattatccccttaatagagaacgctatgATGGGAATTAGCCATGGTCC	For *queE* deletion
queE-P2	agtcacagacggcatcggcaggccgtctgtaagattagtaatgcatcaTGTAGGCTGGAGCTGCTTCG	
queE-F	atgcagtacccgattaacga	For verifiying *queE* deletion
queE-R	atgcgtttgcatagacagcc	
queE-U	ggcgtaatcctgcttttacc	
queE-D	cagcaccacattctaccaac	
ugtL-P1	tccagttgggcgataaaattataaaaacctgcgaggaggctcaaaatgATGGGAATTAGCCATGGTCC	For *ugtL* deletion
ugtL-P2	tggtcatagccattattcagtaagaccgcaggttgcagcggcggatcaTGTAGGCTGGAGCTGCTTCG	
ugtL-F	tgcagtcagaatggctaagg	For verifiying *ugtL* deletion
ugtL-R	tcacgggcgtgaagaaacat	
ugtL-U	ggcagaaggccaatactgat	
ugtL-D	ttgtcggcgataatgcactg	
prfC-P1	caacatgcgtggtcaaacgccgcgcttactcaagaagatttgactatgATGGGAATTAGCCATGGTCC	For *prfC* deletion
prfC-P2	cagggaaagtacgaaggcgcggcggatgccgcgcccggaaagggatcaTGTAGGCTGGAGCTGCTTCG	
prfC-F	atgacgttgtctccttatttgca	For verifiying *prfC* deletion
prfC-R	ttttacggaactgaacgtcagg	
prfC-U	tgtcgacctgctggcttaac	
prfC-D	gccacccttccgtttcagtt	
sulA-P1	ggggtactgtatgaatatacagtaactcacagggctggattgattatgATGGGAATTAGCCATGGTCC	For *sulA* deletion
sulA-P2	gatacgttcgcgtaaaaaaaagtccaggataaatcctaattttacttaTGTAGGCTGGAGCTGCTTCG	
sulA-F	atgtacacttcaggttatgcaa	For verifiying *sulA* deletion
sulA-R	attagagtgaatttttagcccg	
sulA-U	agagtgctctcataaaattcct	
sulA-D	tggaaacttacaagtgtgaact	
dksA-P1	tcccccgaacatggggatcgatagtgcgtgttaaggagaagcaacatgATGGGAATTAGCCATGGTCC	For *dksA* deletion
dksA-P2	gcgggaaaactcccgcctgtcataaatagggtagaaacgaacgggattaTGTAGGCTGGAGCTGCTTCG	
dksA-F	atgcaagaagggcaaaaccgt	For verifiying *dksA* deletion
dksA-R	ttaacccgccatctgtttttc	
dksA-U	gtagtggaataacagcctgat	
dksA-D	ttaacgagccgaaatgcagtt	
rpsL K42T oligo-1	AAACaaactttacgcagtgcggagttcggttttG	For S-*rpsL* K42T contruction and sequencing
rpsL K42T oligo-2	AAAACaaaaccgaactccgcactgcgtaaagttt	
rpsL K42T oligo-3	AAACTTTACGCAGTGCGGAGTTCGGTTTTGTAGGAGTGGTAGTATATACACGAG	
rpsL-up	agacagtgtcgtcagggttga	
rpsL-down	ttacgctgaccaatgacgcga	
FtsZ-YFP-IF	gaaagaggagaaatctgcagatgtttgaacctatggaactaacca	For pZS-P*araBAD*-FtsZ-YFP plasmid construction
FtsZ-YFP-IR	tcacctttgctcatggtaccatcagcttgcttacgcaggaatg	
araBAD-FtsZ-IF	ccatccatgggaattcaaagaggagaaatctgcagatg	
araBAD-FtsZ-IR	tcagctaattaagcttctatttatacagttcgtccataccg	
HisL-eCFP-IF	CGCACTGACCGAATTCATGACACGCGTTCAATTTAAACAC	For HisL translational fusion
HisL-eCFP-IR	TCAGCTAATTAAGCTTTTATTTATACAGTTCATCCATGCCC	
m-tet-HisL-eCFP-IF	CTGATAGGGACTCGAGATTTGTCCTACTCAGGAGAGCGTTC	
m-tet-HisL-eCFP-IR	CTTCACCTCGATTCGAATTTGTTTAACTTTAAGAAGG	
hisL-Nano-IF	gataacaattgaattcctcgagttgctttaaggcgtaaa	For *hisL* transcriptional fusion
hisL-Nano-IR	tcttctaaagtaaaaaccatctgcaggcaattctcgtga	
tet-m-*hisL*-IF	CTTCACCTCGATTCGAATTGCTTTAAGGCGTAAAAGTGGT	
tet-m-*hisL*-IR	CTGATAGGGACTCGAGATTTGTCCTACTCAGGAGAGCGTTC	
hisL-TTG-F	ataagcattcatcggaatttttTtgacacgcg	For *hisL* TTG point mutaton
hisL-TTG-R	cgcgtgtcaAaaaaattccgatgaatgcttat	
rpoD-F	AATGCTCCGTTGCCGAATA	For RT-qPCR detection and normalizaton
rpoD-R	TCGACAAAGCCGGTGATAAG	
hisG-F	CCTGCGTGAAGTCGAAGTTATC	
hisG-R	CCTGAATACGGGTCAGCAATTTA	
5S probe	[Biotin] CTACGGCGTTTCACTTCTGA	For tRNA His detection and normalizaton
His probe	[Biotin] GGGTGGCTAATGGGATTCGA	

Movie S1. Regrowth of filamentous *rpsL *Salmonella* after acid stress removal.** Related to Figure 8A.

References

1. Dai, X., Zhu, M., Warren, M., Balakrishnan, R., Patsalo, V., Okano, H., Williamson, J.R., Fredrick, K., Wang, Y.P. and Hwa, T. (2016) Reduction of translating ribosomes enables *Escherichia coli* to maintain elongation rates during slow growth. *Nat Microbiol*, **2**, 16231.

2. Datsenko, K.A. and Wanner, B.L. (2000) One-step inactivation of chromosomal genes in *Escherichia coli* K-12 using PCR products. *Proc Natl Acad Sci USA*, **97**, 6640-6645.

3. Adeleye, S.A. and Yadavalli, S.S. (2024) Queuosine biosynthetic enzyme, QueE moonlights as a cell division regulator. *PLoS Genet*, **20**, e1011287.

4. Hou, W., Harjono, V., Harvey, A.T., Subramaniam, A.R. and Zid, B.M. (2023) Quantification of elongation stalls and impact on gene expression in yeast. *RNA*, **29**, 1928-1938.
